# ABySS 2.0: resource-efficient assembly of large genomes using a Bloom filter

**DOI:** 10.1101/gr.214346.116

**Published:** 2017-05

**Authors:** Shaun D. Jackman, Benjamin P. Vandervalk, Hamid Mohamadi, Justin Chu, Sarah Yeo, S. Austin Hammond, Golnaz Jahesh, Hamza Khan, Lauren Coombe, Rene L. Warren, Inanc Birol

**Affiliations:** Canada's Michael Smith Genome Sciences Centre, British Columbia Cancer Agency, Vancouver, British Columbia, V5Z 4S6, Canada

## Abstract

The assembly of DNA sequences de novo is fundamental to genomics research. It is the first of many steps toward elucidating and characterizing whole genomes. Downstream applications, including analysis of genomic variation between species, between or within individuals critically depend on robustly assembled sequences. In the span of a single decade, the sequence throughput of leading DNA sequencing instruments has increased drastically, and coupled with established and planned large-scale, personalized medicine initiatives to sequence genomes in the thousands and even millions, the development of efficient, scalable and accurate bioinformatics tools for producing high-quality reference draft genomes is timely. With ABySS 1.0, we originally showed that assembling the human genome using short 50-bp sequencing reads was possible by aggregating the half terabyte of compute memory needed over several computers using a standardized message-passing system (MPI). We present here its redesign, which departs from MPI and instead implements algorithms that employ a Bloom filter, a probabilistic data structure, to represent a de Bruijn graph and reduce memory requirements. We benchmarked ABySS 2.0 human genome assembly using a Genome in a Bottle data set of 250-bp Illumina paired-end and 6-kbp mate-pair libraries from a single individual. Our assembly yielded a NG50 (NGA50) scaffold contiguity of 3.5 (3.0) Mbp using <35 GB of RAM. This is a modest memory requirement by today's standards and is often available on a single computer. We also investigate the use of BioNano Genomics and 10x Genomics’ Chromium data to further improve the scaffold NG50 (NGA50) of this assembly to 42 (15) Mbp.

De novo genome assembly remains a challenging problem, especially for large and complex genomes. The problem refers to reconstructing the chromosome sequence(s) for a genome from sequencing reads, which are orders of magnitude shorter than the target genome (Nagarajan and Pop 2013). In practice, current state-of-the-art assemblers do not fully reconstruct the chromosome sequences but rather reduce the input sequencing reads to a smaller number of nonredundant, more contiguous sequences (contigs). If further linkage information is available, such as in the form of paired-end reads or physical maps, these contigs may be ordered and oriented with respect to each other and reported as scaffolds, where there may be undetermined sequences (represented as “N”s) between contigs. The quality of returned contigs and scaffolds are conventionally measured by the contiguity of the assembled sequences. Often assembly algorithms are also validated using data from resequencing experiments, where assembled sequences are compared against a reference genome for their correctness in addition to their contiguity (Gurevich et al. 2013).

Performance of sequence assembly algorithms is closely coupled with the sequencing technology used and the quality of the data they generate, with highly accurate long reads always being desirable. However, the genomics research landscape, especially cancer genomics studies, has been heavily dominated by the high-throughput sequencing platforms from Illumina. Although longer (albeit noisier) sequences from Pacific Biosciences instruments are proven to yield high-quality de novo human genome assemblies (Chaisson et al. 2014; Pendleton et al. 2015), they come at a higher price relative to Illumina reads. The newer long-read instruments from Oxford Nanopore Technologies do not yet have the necessary throughput or data quality to be of utility in human genomics studies. As a result, most large cohort projects, as well as price-sensitive personalized medicine applications, still use the Illumina platforms.

Recently, new sequencing technologies have been introduced that combine long-range linkage information with the strengths of existing Illumina short-read technologies. The Chromium platform from 10x Genomics generates sequencing libraries that localize sequence information on DNA fragments that are >100 kb long. The technology employs microfluidics to isolate large DNA fragments in partitions containing sequencing primers and a unique barcode, preparing a library that is compatible with Illumina paired-end sequencing (Weisenfeld et al. 2017). Another recent technology for long-range linkage information is the optical mapping platform from BioNano Genomics. It has previously been demonstrated in the Human Genome Project (International Human Genome Sequencing Consortium 2001) and other pioneering de novo sequencing projects that linkage information from a physical map is very valuable in building highly contiguous assemblies. In this article, we show that 10x Genomics data and BioNano Genomics data can be used in combination to substantially improve the contiguity of a de novo assembly.

Of particular interest in this study is resequencing data from human genome studies. The approach of de novo assembly of data from these experiments prior to comparison to a reference sequence is a valuable approach in detecting structural variants between individuals or between tumor and normal genomes (Mose et al. 2014; Li 2015b). Even though it is substantially more computationally intensive to analyze sequencing data by assembling the reads first, the specificity gains and resulting savings in event verification efforts may justify the choice. However, when working with mammalian-size genome data, de novo assembly is often plagued with long assembly run times and prohibitively large memory requirements—resource usages that warrant improvements.

In this domain, ABySS 1.0 was the first scalable de novo assembly tool that could assemble a human genome, using short reads from a high-throughput sequencing platform (Simpson et al. 2009). However, the feat required aggregating a large amount of memory distributed across a number of compute nodes communicating through the message-passing interface (MPI) protocol. Although this enabling technology found applications in many large cancer cohort studies (Yip et al. 2011; Roberts et al. 2012; Ley et al. 2013; Morin et al. 2013; Pugh et al. 2013), its large memory consumption constituted a substantial bottleneck. This issue was not unique to ABySS 1.0, with popular algorithms such as SOAPdenovo2 (Luo et al. 2012) and DISCOVAR de novo requiring >600 GB to assemble a typical human data set. Some noteworthy assembly algorithms that have been developed to reduce memory requirements include the following: (1) SGA (Simpson and Durbin 2011), which uses the Burrows-Wheeler transform to compress and index sequencing data; (2) Minia (Chikhi and Rizk 2013), which uses a Bloom filter (Bloom 1970) to represent the de Bruijn graph; (3) BCALM 2 (Chikhi et al. 2016), which employs minimizer hashing (Chikhi et al. 2014) to partition the de Bruijn graph; and (4) MEGAHIT (Li et al. 2016), which employs the Succinct de Bruijn graph data structure of Bowe et al. (2012).

In this article, we describe the implementation of ABySS 2.0, which reduces memory requirements for de novo assembly by an order of magnitude, while achieving results competitive with existing assemblers. ABySS 2.0 follows the model of Minia, wherein a probabilistic Bloom filter representation is used to encode the de Bruijn graph. We compare the performance of ABySS 2.0 against the latest version of ABySS 1.0, as well as two other scalable assembly pipelines that include a scaffolding stage: SOAPdenovo2 and SGA. We note that there are other algorithms that can build contigs without scaffolding, and we include comparison to DISCOVAR de novo, MEGAHIT, Minia, and BCALM 2, scaffolding the contigs of DISCOVAR de novo using the scaffolding tools BESST (Sahlin et al. 2016), LINKS (Warren et al. 2015), and the ABySS scaffolding algorithm. We also demonstrate how long-range linkage information from Chromium reads and BioNano maps may improve scaffold contiguity of draft genome assemblies.

## Results

### Overview of ABySS 2.0 assembly algorithm

ABySS 1.0 is a multistage de novo assembly pipeline consisting of unitig, contig, and scaffold stages. At the unitig stage, we perform the initial assembly of sequences according to the de Bruijn graph assembly paradigm (Pevzner et al. 2001). At the contig stage, we align the paired-end reads to the unitigs and use the pairing information to orient and merge overlapping unitigs. At the scaffold stage, we align the mate-pair reads to the contigs to orient and join them into scaffolds, inserting runs of “N” characters at gaps in coverage and for unresolved repeats. The most resource-intensive stage of ABySS 1.0 is the unitig (de Bruijn graph) assembly stage and is also its peak memory requirement. This stage of the pipeline loads the full set of *k*-mers from the input sequencing reads into a hash table and stores auxiliary data for each *k*-mer such as the number of *k*-mer occurrences in the reads and the presence/absence of possible neighbor *k*-mers in the de Bruijn graph. ABySS 1.0 addresses the large memory requirement by implementing a distributed version of the de Bruijn graph assembly approach, wherein the hash table of *k*-mers is split across cluster nodes, and communication between nodes occurs via the MPI standard. By these means, ABySS 1.0 enables the assembly of large genomes on clusters of commodity hardware. For example, ABySS 1.0 was used to assemble the 20-Gbp white spruce genome with 115 cluster nodes and ∼4.3 TB of aggregate memory (Birol et al. 2013).

**Figure 1.**
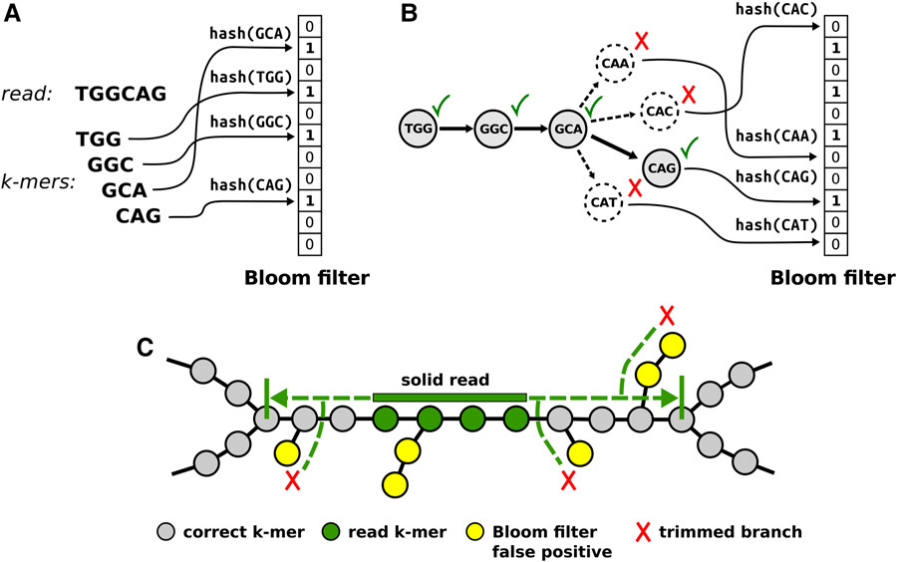
Overview of the ABySS 2.0 assembly algorithm. (*A*) *k*-mers from each input sequencing read are loaded into the Bloom filter by computing the hash values of each *k*-mer sequence and setting the corresponding bit in the Bloom filter. For clarity, we show a Bloom filter that uses a single hash function; in practice, multiple bit positions are set for each *k*-mer using multiple independent hash functions. (*B*) A path in the de Bruijn graph is traversed by repeatedly querying for possible successor *k*-mers and advancing to the successor(s) that are found in the Bloom filter. Each possible successor corresponds to single-base extension of the current *k*-mer by “A,” “C,” “G,” or “T.” (*C*) ABySS 2.0 builds unitig sequences by extending solid reads *left* and *right* within the de Bruijn graph. A solid read is a read that consists entirely of *k*-mers with an occurrence count greater or equal to a user-specified threshold (solid *k*-mers); the optimum minimum occurrence threshold is typically in the range of two to four. Extension of a solid read is halted when either a branching point or a dead end in the de Bruijn graph is encountered. A look-ahead algorithm is employed to detect and ignore short branches caused by Bloom filter false positives and/or recurrent read errors.

The main innovation of ABySS 2.0 is a Bloom filter-based implementation of the unitig assembly stage, and it reduces the overall memory requirements by an order of magnitude, enabling assembly of large genomes on a single machine. A Bloom filter (Bloom 1970) is a compact data structure for representing a set of elements that supports two operations: (1) inserting an element into the set, and (2) querying for the presence of an element in the set. In the context of Bloom filter-based de Bruijn graph assembly algorithms, the elements of the set are the *k*-mers of the input sequencing reads. The Bloom filter data structure consists of a bit vector and one or more hash functions, where the hash functions map each *k*-mer to a corresponding set of positions within the bit vector (Fig. 1A); we refer to this set of bit positions as the *bit signature* for the *k*-mer. A *k*-mer is inserted into the Bloom filter by setting the positions of its bit signature to one and is queried by testing if all positions of its bit signature are one. While a Bloom filter provides a very memory-efficient means of representing a set, it has the caveat that it can generate *false positives* when the bit signatures of different *k*-mers overlap by chance. In the context of our application, this means that a certain fraction of *k*-mer queries will return true even though the *k*-mers do not exist in the input sequencing data. The false-positive rate (FPR) for a Bloom filter (Bloom 1970) can be estimated using

(1) $$\text{FPR}={\left(1-{\left(1-\frac{1}{m}\right)}^{\mathrm{hn}}\right)}^{h}\approx {\left(1-e^{-(\mathrm{hn}/m)}\right)}^{h}$$

where *m* is the Bloom filter in bits, *h* is the number of hash functions, and *n* is the number of distinct *k*-mers in the data. Handling false positives was the main design challenge of ABySS 2.0, and we discuss the issue in further detail in Methods.

During unitig assembly, two passes are made through the input sequencing reads. In the first pass, we extract the *k*-mers from the reads and load them into a Bloom filter (Fig. 1A). To filter out the majority of *k*-mers caused by sequencing errors, we discard all *k*-mers with an occurrence count below a user-specified threshold, typically in the range of two to four. We refer to the retained *k*-mers as *solid k-mers*. In the second pass through the reads, we identify reads that consist entirely of solid *k*-mers, which we term *solid reads*, and extend them left and right within the de Bruijn graph to create unitigs (Fig. 1C). During read extension, we adopt the same approach to graph traversal as originally described for Minia (Chikhi and Rizk 2013). Since only the nodes (*k*-mers) of the de Bruijn graph are stored in the Bloom filter and not the edges, we query all four possible *k*-mers neighboring the current *k*-mer during each step of graph traversal. This step enables us to discover outgoing edges (Fig. 1B). We note that during the read extension phase of assembly, it is possible for multiple solid reads to result in the same unitig. To avoid such duplicate sequences, we use an additional tracking Bloom filter to record *k*-mers included in previous unitigs, and a solid read is only extended if it has at least one *k*-mer that is not already in the tracking Bloom filter.

### Effect of Bloom filter FPR

In the context of de Bruijn graph assembly, Bloom filter false positives have the effect of adding to the graph *k*-mers that are not present in the input sequencing reads. To address this issue, we have implemented a look-ahead mechanism to remove such *k*-mers from the graph, as described in the Methods. However, in order to confirm that Bloom filter false positives do not cause assembly artifacts and to better understand the relationship between Bloom filter FPR, assembly quality, RAM usage, and running time, we conducted the following experiment.

**Figure 2.**
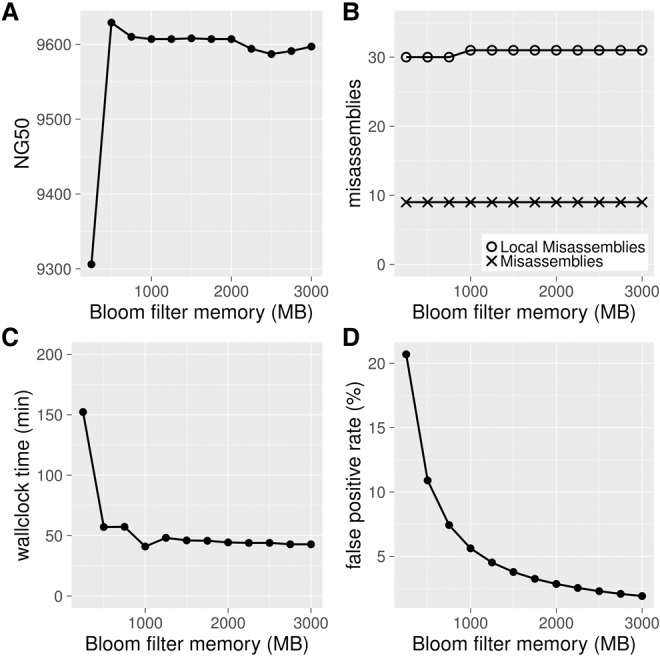
Effect of Bloom filter memory allocation on ABySS 2.0 assemblies of the *C. elegans* DRR008444 data set. (*A*) The assembly contiguity (NG50) remains stable in the neighborhood of 9600 bp as the Bloom filter allocation decreases from 3000 MB of 500 MB but drops sharply as the allocation is further decreased from 500 to 250 MB. (*B*) The number of major misassemblies (9) and local misassemblies (30–31) reported by QUAST remains stable as the Bloom filter allocation is decreased from 3000 to 250 MB. (*C*) The assembly wall-clock time increases gradually as the Bloom filter allocation is decreased from 3000 to 500 MB but rises sharply from 57 to 152 min when the allocation is further decreased from 500 to 250 MB. (*D*) The relationship between Bloom filter false-positive rate and the Bloom filter memory allocation. From these results, we conclude that a Bloom filter FPR in the range of 5%–10% provides a good balance between assembly time and memory usage, without any detrimental effect on assembly quality.

By using the *Caenorhabditis elegans* data set DRR008444, we conducted assemblies with a range of Bloom filter FPRs and measured the resulting NG50 length metric, number of misassemblies, and wall-clock time (Fig. 2). We note that the Bloom filter FPR is not a directly tunable parameter of ABySS 2.0. Instead, we controlled the FPR indirectly by changing the Bloom filter size from 250 to 3000 MB, with a step size of 250 MB. Further details of the experimental setup are provided in “Effect of Bloom Filter Positive Rate” in the Supplemental Material. In Figure 2A, we observe that the NG50 remains stable in the neighborhood of 9600 bp as the Bloom filter allocation decreases from 3000 to 500 MB, corresponding to FPR values of 1.91% and 10.9%, respectively, but drops sharply when Bloom filter allocation is decreased further from 500 to 250 MB (FPR 10.9% and 20.7%, respectively). Similarly, the number of major misassemblies (9) and local misassemblies (30–31) reported by QUAST 3.2 remains stable as the Bloom filter allocation is decreased from 3000 to 250 MB (Fig. 2B). Additional QUAST metrics indicate that genome assemblies are of similar quality with a Bloom filter allocation as low as 500 MB (detailed in Supplemental Figs. S1–S3; Supplemental Tables S1–S3). Finally, in Figure 2C we observe that the run time of ABySS 2.0 is inversely related to Bloom filter size. This behavior is due to the use of a look-ahead algorithm to trim false branches from the de Bruijn graph, as described in the Methods and depicted in Figure 1C. Run time increases gradually as the Bloom filter allocation decreases from 3000 to 500 MB but rises sharply from 57 to 152 min when the allocation is further decreased from 500 to 250 MB. These plots demonstrate a trade-off between memory usage and run time, with an FPR in the range of 5%–10% giving both good memory usage and time performance. It also indicates that any FPR <20% has no adverse effects on assembly quality, considering both contiguity and correctness.

Most false-positive *k*-mers result in a tip that is pruned by the look-ahead algorithm. In a standard de Bruijn graph, two *k*-mers that occur at distant locations in the genome but, coincidentally, share an overlap of *k* − 1 nucleotides cause a branch in the de Bruijn graph, stopping the assembly of a contig at that branching point. The false-positive *k*-mers of a Bloom filter de Bruijn graph can make a connection between two *k*-mers that overlap by fewer than *k* − 1 nucleotides. Such a chance connection similarly creates a branching point causing the contig to come to a premature end. The probability of such a chance connection decreases exponentially with a decreasing overlap, FPR^*k*−1−*o*^, where *o* is the amount of overlap between the two *k*-mers. If these chance connections occurred frequently, we would expect that varying the size of the Bloom filter and thus the FPR to significantly affect the contiguity of the assembly. However, we show empirically in Figure 2A that the contiguity of the assembly is largely insensitive to the FPR, and we surmise that these chance connections occur infrequently.

### Assembler comparison

To assess the performance of ABySS 2.0, we compared it with other leading assemblers for large genomes: ABySS 1.0 (Simpson et al. 2009), BCALM 2 (Chikhi et al. 2016), DISCOVAR de novo, MEGAHIT (Li et al. 2016), Minia (Chikhi and Rizk 2013), SGA (Simpson and Durbin 2011), and SOAPdenovo2 (Luo et al. 2012). We note that DISCOVAR de novo is the whole-genome de novo assembly successor to DISCOVAR (Weisenfeld et al. 2014). We conducted our comparison using a recent, publicly available human short-read data set provided by the Genome in a Bottle (Zook et al. 2016) project. The NIST HG004 (Coriell cell line NA24143) data were chosen for its deep 70× coverage of Illumina short-read (paired-end 250 bp) data and the availability of sequences from other platforms, including a 175× physical coverage mate-pair data set (after trimming), 10x Genomics Chromium data, and BioNano optical mapping data. We note that paired-end 250-bp sequencing data from an Illumina HiSeq 2500 in rapid-run mode is currently roughly double the cost per base of paired-end 125-bp sequencing data on the same machine in high-throughput mode (http://bit.ly/hiseq2500, http://bit.ly/cornell-price-list).

Each of the assemblers in the comparison was chosen due to its significant contributions toward the goal of scalable de novo assembly. The previous version of ABySS facilitates large genome assemblies by distributing the de Bruijn graph across cluster nodes and was the first software to assemble a human genome from short reads. The BCALM 2 assembler introduces a novel method for partitioning the de Bruijn graph using minimizer hashing, which enables subsets of the graph to be assembled iteratively or in parallel. DISCOVAR de novo is a recent de Bruijn graph assembler for large genomes. MEGAHIT utilizes a data structure called a succinct de Bruijn graph (Bowe et al. 2012) to reduce the memory requirements for de Bruijn graph assembly. Minia is the first assembler to employ a Bloom filter representation of the de Bruijn graph and uses a novel algorithm for eliminating Bloom filter false positives. SGA demonstrates the use of an FM-index (Simpson and Durbin 2011) as the core data structure for assembly, enabling detection of variable-length overlaps between reads with a low memory footprint. In addition to the aforementioned assemblers, we also attempted to include ALLPATHS-LG 52488 (Gnerre et al. 2010) and MaSuRCA 3.1.3 (Zimin et al. 2013); however, we were unable to run these assemblers to completion on the HG004 data set (for details, see “Assembler Scripts and Configuration Files” in Supplemental Material). For the majority of assemblers, we conducted assemblies across a range of *k*-mer sizes and selected a single assembly for inclusion in the comparison that represented the best tradeoff between maximizing contiguity (NG50 and NGA50) and minimizing alignment breakpoints with respect to reference genome GRCh38 (Supplemental Fig. S5; Supplemental Tables S5–S9). Further details regarding *k*-mer size optimization are described in “*K*-mer Size Sweeps” in the Supplemental Material.

**Figure 3.**
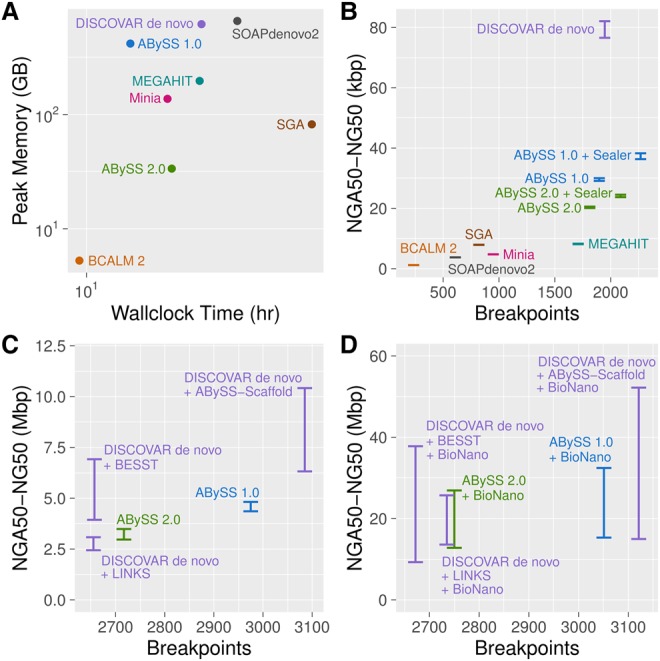
De novo assembly results for Genome in a Bottle HG004 human genome short-read data with ABySS 1.0, ABySS 2.0, BCALM 2, DISCOVAR de novo, MEGAHIT, Minia, SOAPdenovo2, and SGA. To enable comparison with ABySS, the DISCOVAR de novo assembly was scaffolded with third-party scaffolders ABySS-Scaffold, LINKS (Warren et al. 2015), and BESST (Sahlin et al. 2016). For panels *B*–*D*, on the *y*-axes we show the range of NGA50 to NG50 to indicate uncertainty caused by real genomic variants between individual HG004 and the reference genome (GRCh38). On the *x*-axes, we show the number of breakpoints that occurred when aligning the sequences to the reference genome. (*A*) Peak memory usage and wall-clock time for the assemblers. (*B*) Contiguity and correctness metrics for contig sequences. (*C*) Contiguity and correctness metrics after scaffolding with mate-pair (MPET) reads. The SOAPdenovo2 result for this plot was excluded as an outlier with an NGA50 (NG50) value of 103 kbp (172 kbp) and 10,610 breakpoints. (*D*) Contiguity and correctness metrics after further scaffolding with BioNano optical mapping data, using BioNano's hybrid scaffolding pipeline.

In Figure 3A and Table 1, we compare the peak RAM usage and wall-clock time of the assemblers. All assemblies from Figure 3 were benchmarked on a server with 2.5 TB of RAM and four Xeon E7-8867 v3 CPUs running at 2.50 GHz, providing a total of 64 cores. Memory usage and run time of the assemblers varied from 5 GB (BCALM 2) to 659 GB (SOAPdenovo2) and 9 h (BCALM 2) to 65 h (SGA). The tools that represent the de Bruijn graph succinctly—ABySS 2.0, MEGAHIT, Minia, and SGA—had memory footprints many times smaller than ABySS 1.0, DISCOVAR de novo, and SOAPdenovo2. BCALM 2 achieved both the smallest memory footprint, by virtue of its novel partitioning strategy to constructing the de Bruijn graph, and completed the assembly in 9 h, 8 h of which was spent counting *k*-mers with DSK (Rizk et al. 2013). DISCOVAR de novo, which achieved the best sequence contiguity, required 618 GB of memory and 26 h to complete, and SOAPdenovo2 required a similar 659 GB and 35 h. SGA achieves its compact memory usage of 82 GB at the expense of run time, requiring 65 h to complete the assembly. In addition to the results of Figure 3A, we performed further benchmarking of ABySS 1.0 and ABySS 2.0 on other platforms (Supplemental Table S10). Most notably, we ran the ABySS 2.0 assembly on a low-memory machine with 48 GB RAM and 12 CPU cores, with a peak memory usage of 34 GB and a wall-clock time of 80 h.

**Table 1.**
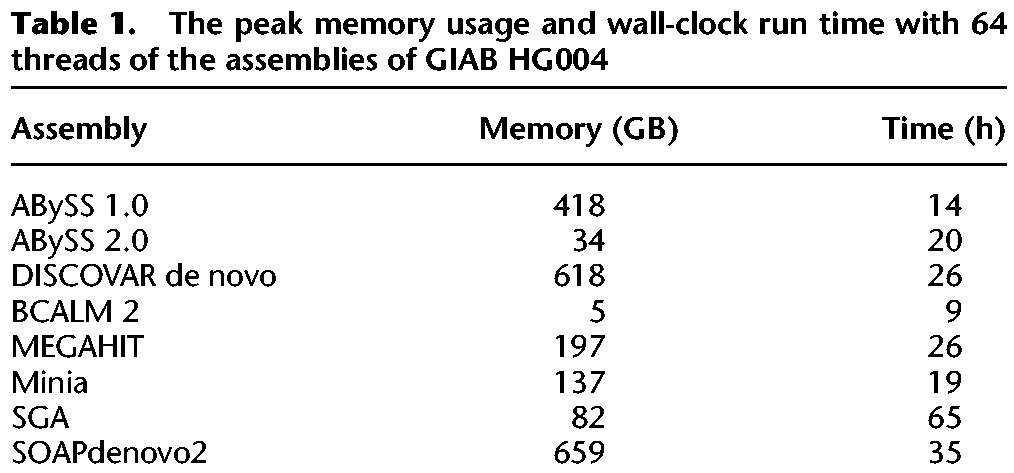
The peak memory usage and wall-clock run time with 64 threads of the assemblies of GIAB HG004

In Figure 3B and Table 2, we compare the contiguity and correctness of the contig sequences generated by the assemblers. To extract contigs from the assemblies, we split the sequences at occurrences of one or more “N” characters. In addition to comparing the contigs produced by each assembler, we included two additional data points (“ABySS 1.0 + Sealer”, “ABySS 2.0 + Sealer”) in Figure 3B to show the contiguity improvement produced by closing scaffold gaps with Sealer (Paulino et al. 2015), prior to splitting the scaffold sequences at “N”s. Further details regarding the Sealer results are provided in “Gap Filling with Sealer” in the Supplemental Material. To assess the contiguity of the contigs, we calculated both NG50 and NGA50 using a genome size of 3,088,269,832 bp. To assess assembly correctness, we counted the number of breakpoints when aligning the contigs to the primary chromosome sequences of the human reference GRCh38. Comparing the NG50 and NGA50 of the contigs, we observe that DISCOVAR de novo achieves the highest sequence contiguity by a factor of approximately two (DISCOVAR de novo NG50 of 82 kbp vs. ABySS 1.0 + Sealer NG50 of 38 kbp), although its memory use is the second largest, exceeded only by SOAPdenovo2. We note that the NG50 of the ABySS 1.0 (30 kbp) and ABySS 2.0 (21 kbp) contigs noticeably exceeds those of BCALM 2 (1 kbp), MEGAHIT (8 kbp), and Minia (5 kbp), primarily due to the additional use of paired-end information in ABySS. We also note that ABySS 2.0 achieves a lower contiguity than ABySS 1.0 (21 kbp vs. 30 kbp). Upon investigation, we conclude that the main cause of this difference is the handling of low coverage regions. Whereas ABySS 1.0 retains all *k*-mers in the de Bruijn graph along with their counts, ABySS 2.0 discards *k*-mers with counts below a user-specified threshold, as discussed in the Methods. To further assess the assemblies, we calculated the percentage of sequence identity and percentage of genome coverage of the contigs aligned to the reference genome. The percentage of identity ranged from 99.5% to 99.8%, the percentage of genome coverage from 93% to 98%, and ABySS 2.0 scored near the upper ends of both measures with 99.7% identity and 96% genome coverage (Supplemental Fig. S4; Supplemental Table S4).

**Table 2.**
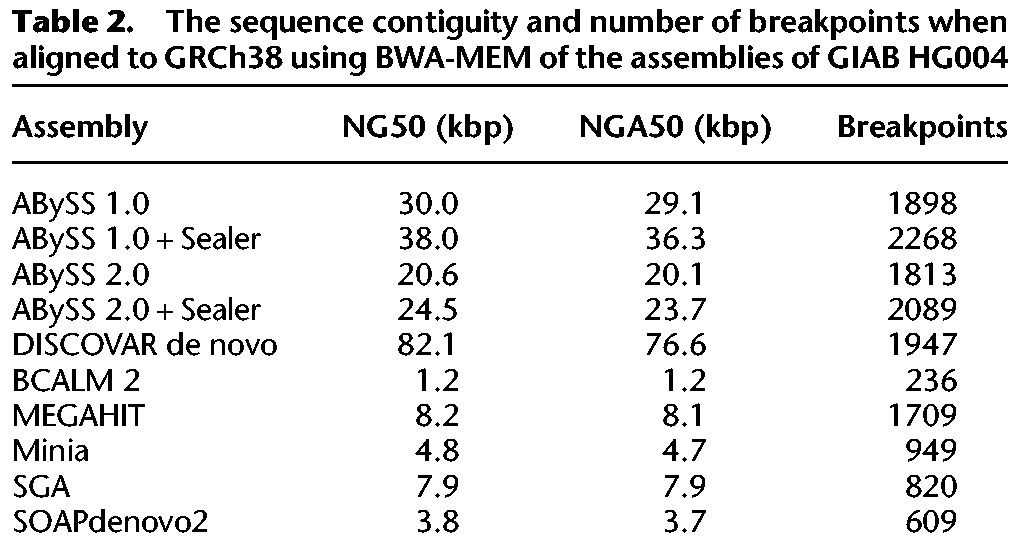
The sequence contiguity and number of breakpoints when aligned to GRCh38 using BWA-MEM of the assemblies of GIAB HG004

In Figure 3C and Table 3, we compare the contiguity and correctness of the assemblies after scaffolding with Illumina mate-pair data. We generally excluded assemblers from this stage of the comparison that did not implement their own scaffolding algorithms. However, in light of the strong contiguity results of DISCOVAR de novo at the contig stage, we chose to scaffold the DISCOVAR de novo contigs with several third-party scaffolders: ABySS-Scaffold (data not shown), LINKS (Warren et al. 2015), and BESST (Sahlin et al. 2016). In comparison to Figure 3B, we note that the NG50 and NGA50 values of the DISCOVAR de novo and ABySS assemblies begin to converge, as do the values for the two versions of ABySS compared. We also note that there are significant differences between the scaffold NG50 and NGA50 length metrics, particularly in the case of the DISCOVAR de novo + ABySS-Scaffold assembly with an NG50 of 10.4 Mbp and NGA50 of 6.3 Mbp. We understand this divergence to be caused by the differing assumptions of the two contiguity metrics. While the NG50 is calculated under the assumption that all sequences are correctly assembled, the NGA50 metric penalizes breakpoints when aligning the sequences to the reference genome. While the NG50 is an overly optimistic metric, the NGA50 is an overly pessimistic metric because certain breakpoints may be attributed to real structural variation between the sequenced individual and the reference genome. For this reason, we show contiguity of the assemblies as a range between NGA50 and NG50, with the true unknown value lying somewhere in between.

**Table 3.**
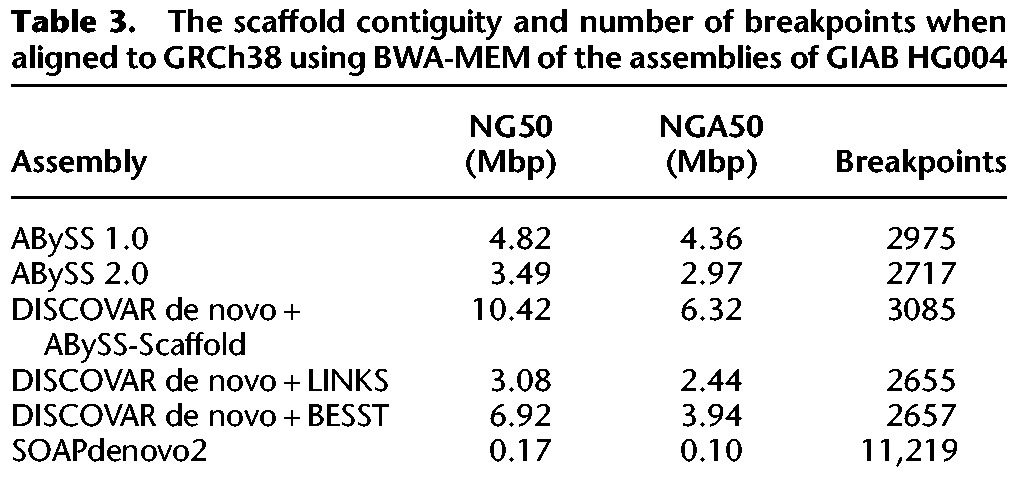
The scaffold contiguity and number of breakpoints when aligned to GRCh38 using BWA-MEM of the assemblies of GIAB HG004

In Figure 3D and Table 4, we show the results after an additional round of scaffolding of the DISCOVAR de novo and ABySS assemblies using the BioNano optical map for individual HG004, as provided by the Genome in a Bottle project. The BioNano protocol generates an optical map of the genome by fluorescently tagging occurrences of a particular endonuclease motif within long DNA molecules, resulting in a barcode-like pattern for each molecule. To perform the scaffolding, the BioNano software generates an analogous set of barcode patterns in silico for the sequences of the input assembly and then aligns the two sets of bar codes. Applying BioNano scaffolding to the mate-pair–scaffolded sequences improved the NG50 by a factor of five or more across all assemblies, with NG50 reaching 52 Mbp with DISCOVAR de novo + ABySS-Scaffold + BioNano. We observe that the distance between the NG50 and NGA50 values grows even larger at this stage of scaffolding, which we surmise is caused by a greater likelihood of encountering real sequence variation between the sequenced individual and the reference genome.

**Table 4.**
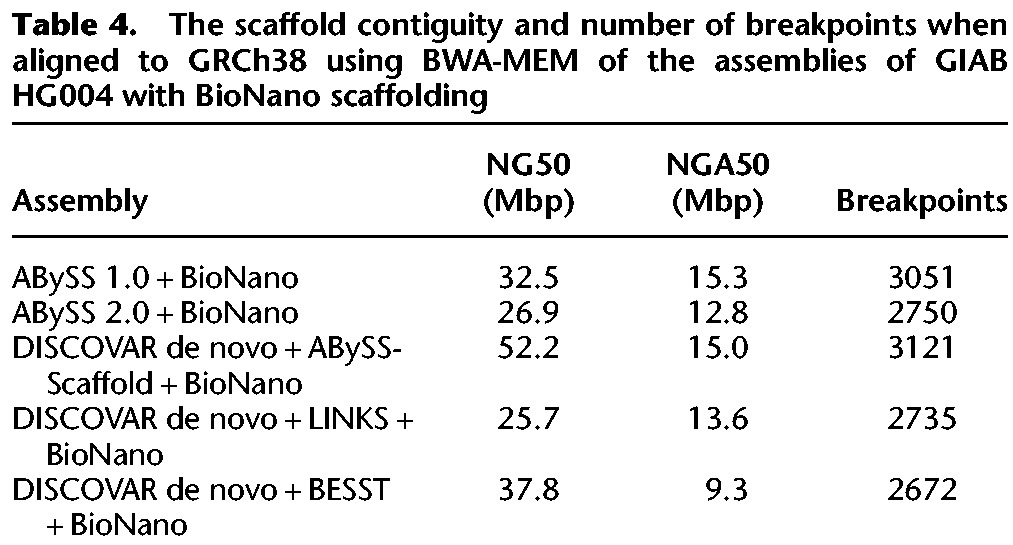
The scaffold contiguity and number of breakpoints when aligned to GRCh38 using BWA-MEM of the assemblies of GIAB HG004 with BioNano scaffolding

**Figure 4.**
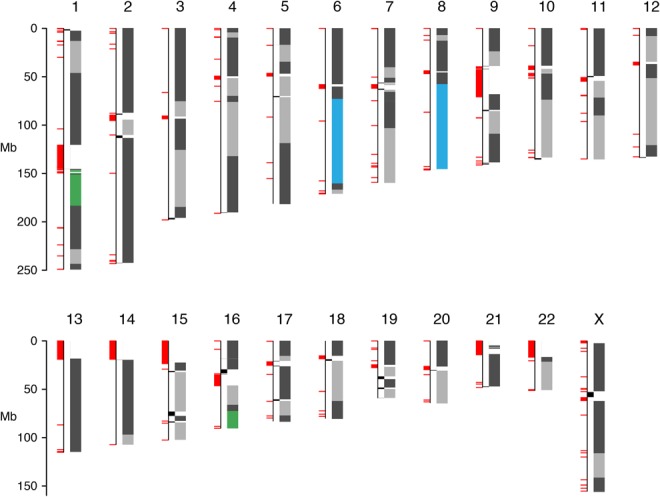
Contigs from the 89 scaffolds >3.2 Mbp that compose 90% of the genome are aligned to GRCh38 using BWA-MEM. Contigs from the same scaffold are shown in the same shade of gray, and alternating shades of light and dark gray are used to distinguish between contigs from different scaffolds. Two translocations, t(1;16) and t(6;8), are shown in green and blue. The segments of the genome that are not covered by alignments of the largest 89 scaffolds are shown offset in black. Gaps in the reference genome, including centromeres and other heterochromatin, are shown in red.

**Figure 5.**
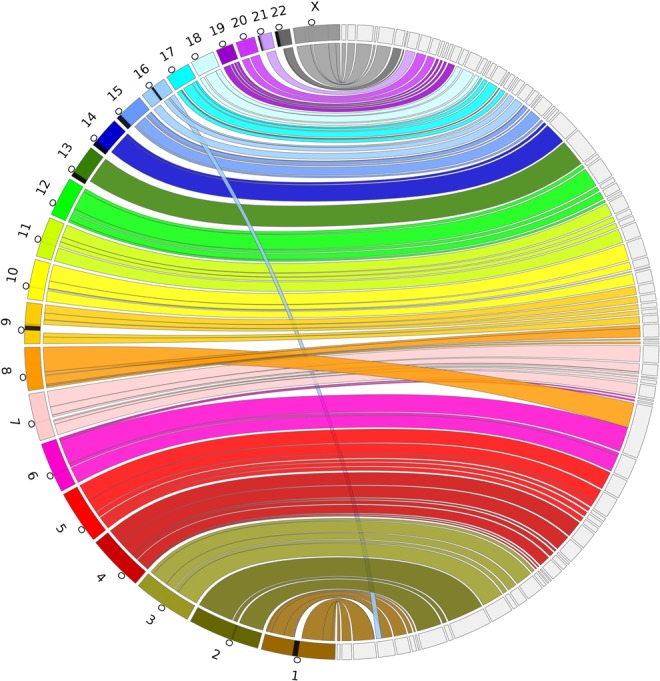
A Circos (Krzywinski et al. 2009) assembly consistency plot. Scaftigs from the largest 89 scaffolds that compose 90% of the genome are aligned to GRCh38 using BWA-MEM. GRCh38 chromosomes are displayed on the *left* and the scaffolds on the *right*. Connections show the aligned regions between the genome and scaffolds. Contigs are included as a part of the same region if they are within 1 Mbp on either side of the connection, and regions <100 kbp are not shown. The black regions on the chromosomes indicate gaps in the reference and the circles indicate the centromere location on each chromosome.

Given the aforementioned limitations of our breakpoint metric for assessing assembly correctness, we additionally performed manual checks for assembly correctness. To this end, we investigated large-scale misassemblies (>10 Mb) and found only two major events within our ABySS 2.0 + BioNano scaffolds (Figs. 4, 5). One of these large-scale events between Chromosomes 1 and 16 was identified in every assembly (Supplemental Figs. S7–S12), which indicates that the event may be a real structural variant with respect to the reference human genome GRCh38. The other large scale event between Chromosomes 6 and 8 is interestingly also found in the DISCOVAR de novo + BESST + BioNano assembly (Supplemental Fig. S11), despite having fewer breakpoints and using an independent methodology. This suggests that the relative correctness of the ABySS 2.0 + BioNano assembly is on par with that of other assemblies.

### Scaffolding with Chromium data

As the final step of our ABySS 2.0 assembly, we used the 10x Genomics Chromium data available for individual HG004 to further scaffold the BioNano assembly. The Chromium sequencing platform augments existing short-read technologies by labeling reads that originate from the same long DNA molecule with a shared barcode sequence. This labeling is achieved during library preparation by isolating long DNA molecules into droplets alongside gel beads containing the barcoding oligos. The barcodes added by the Chromium protocol provide additional long-range grouping information for the short reads, which can be leveraged for scaffolding and other bioinformatics applications, such as phasing sequence variants.

To scaffold our assembly with the Chromium data, we developed ARCS (Yeo et al. 2017). Briefly, we aligned the Chromium linked reads to the input BioNano scaffolds with BWA-MEM and recorded the barcodes of the reads that aligned to each scaffold. As we were only interested in the barcodes that joined scaffolds, we reduced noise by masking the interior portions of the input BioNano scaffolds with “N” characters, preserving only the first/last 30 kbp of sequence in each scaffold, prior to aligning the Chromium reads. By using the information obtained from the read alignments, we constructed a graph representation of the relationships between scaffolds, using nodes to represent scaffolds and edge weights to represent the number of shared barcodes between scaffolds. Finally, we supplied this graph as input to the LINKS (Warren et al. 2015) scaffolding algorithm to identify high-confidence paths within the graph and to output the corresponding scaffolds. Additional information regarding the Chromium scaffolding with ARCS and LINKS is provided in “Assembler Scripts and Configuration Files” in the Supplemental Material.

The Chromium scaffolding increased the scaffold NG50 of our ABySS 2.0 assembly from 26.9 to 41.9 Mbp. At this scale of contiguity, the largest scaffolds represent significant fractions of chromosome arms. In Figure 4, we show the positions on the chromosomes of the 89 scaffolds >3.2 Mbp that compose 90% of the genome. We note that many chromosome arms are reconstructed by one to four large scaffolds, exemplified in Figure 5. We observe two regions indicative of a structural rearrangement and/or misassembly. Interestingly, the t(1;16) translocation is seen in every assembly (Supplemental Figs. S7–S12), and the t(6;8) translocation is also seen in the DISCOVAR de novo + BESST + BioNano assembly (Supplemental Fig. S11).

## Discussion

The ideogram of Figure 4 demonstrates that correct and highly-contiguous de novo assembly of human genomes is possible using current short-read sequencing technologies combined with long-range scaffolding techniques. While each of the scaffolding data types used here (mate-pair, BioNano, Chromium) are capable of increasing assembly contiguity by orders of magnitude on their own, our results demonstrate that these data are even more powerful when used in combination, also demonstrated by Mostovoy et al. (2016). In the human assembly we have described here, each scaffolding step feeds on the success of the previous assembly stages. Longer contig sequences improve the results of mate-pair scaffolding by allowing more mate-pairs to map to the contigs. Longer mate-pair scaffolds improve the BioNano scaffolding by allowing the optical map to align unambiguously to the mate-pair scaffolds; for this reason, BioNano recommends that the input assembly contains sequences of at least 100 kbp. Finally, longer BioNano scaffolds improve the Chromium scaffolding by resolving ambiguities in ordering and orientation of the scaffolds that are difficult to resolve using Chromium data alone.

Another observation that can be made from our assembler comparison is that, in spite of more than a decade of research and development related to de Bruijn graph assemblers, the memory and runtime efficiency of short-read assemblers can still be greatly improved. This issue is particularly important for downstream studies that involve large numbers of de novo assemblies, such as human population studies, cancer genome studies, and clinical applications. The opportunity for improving the throughput of de novo assemblies is evident when comparing novel de Bruijn graph implementations such as Minia and BCALM 2 against more mature assembly pipelines such as ABySS 1.0 and DISCOVAR de novo (Fig. 3A). For example, the BCALM 2 assembly used only 5 GB RAM and 9 h to run, whereas the DISCOVAR de novo assembly used >600 GB of RAM and over a day to run. While Minia and BCALM 2 did not match the results of ABySS and DISCOVAR de novo in terms of assembly contiguity (Fig. 3B), we posit that this is due to the limited error removal of the implementations and not a fundamental limitation of the algorithms themselves. In the case of Minia, this hypothesis is borne out by the results of ABySS 2.0 (Fig. 3A), which employs a Bloom filter-based assembly approach similar to Minia but achieves contiguity results that are on par with DISCOVAR de novo and ABySS 1.0.

The assembly of long reads has yielded highly contiguous genome assemblies of human (Pendleton et al. 2015; Chin et al. 2016) and other organisms with sequence contiguity in the megabase range. Long-read sequencing comes, however, at a cost premium. For applications that are cost-sensitive, such as sequencing for diagnostic medicine, algorithms that exploit high-throughput short-read sequencing are valuable. We show that megabase scaffolds are achievable using short-read sequencing with one paired-end and one mate-pair library, and scaffolds approaching the size of entire chromosome arms are possible when scaffolding with additional BioNano and/or 10x Genomics data. A remaining challenge for short-read assemblies is to improve their sequence contiguity, which remains in the range of tens of kilobases, significantly shorter than the megabases achieved with the assembly of long-read sequencing.

## Methods

### Bloom filter de Bruijn graph assembly

The first stage of the ABySS 2.0 assembly pipeline is a de Bruijn graph assembler that uses a compact, Bloom filter-based representation of the graph. The use of Bloom filters for de novo assembly was first demonstrated in Minia (Chikhi and Rizk 2013), and ABySS 2.0 builds on many aspects of that approach. The parts of our assembly algorithm that are novel with respect to Minia are (1) the use of *solid reads* to seed contig traversals (explained below), (2) the use of look-ahead for error correction and elimination of Bloom filter false positives rather than a separate data structure, and (3) the use of a new hashing algorithm, ntHash (Mohamadi et al. 2016), designed for processing DNA/RNA sequences efficiently.

We will begin by describing the basic aspects of our assembly algorithm that closely follow Minia, including the Bloom filter representation of the de Bruijn graph and the use of a *cascading Bloom filter* to remove low-occurrence *k*-mers. As in Minia, the first step of the assembly algorithm is to load all *k*-mers from the sequencing reads into a Bloom filter (Fig. 1A). These *k*-mers represent the set of nodes in the de Bruijn graph, but we do not explicitly store the edges representing the *k* − 1 bp overlaps between *k*-mers. Instead, as in Minia, we discover edges at runtime by querying the Bloom filter for the four possible predecessors/successors of the current *k*-mer during the course of a graph traversal (Fig. 1B). Each possible successor (predecessor) corresponds to a single-base extension of the current *k*-mer to the right (left) by “A,” “C,” “G,” or “T.” Another technique shared with Minia is the use of a cascading Bloom filter to eliminate low-occurrence *k*-mers, the majority of which are caused by sequencing errors (Vandervalk et al. 2015). Briefly, a cascading Bloom filter is a chained array of Bloom filters where each Bloom filter stores *k*-mers with a count that is one higher than the preceding Bloom filter. The procedure for inserting a *k*-mer into a cascading Bloom filter is to query each Bloom filter in succession and to add the *k*-mer to the first Bloom filter where it is not already present. After all *k*-mers from the reads have been inserted, the last Bloom filter in the chain is then kept as the set of *solid k-mers* and the preceding Bloom filters are discarded. We note that ABySS 2.0 assigns equal sizes to each Bloom filter in the cascading chain, and so using *c* cascading Bloom filter levels effectively multiplies the peak memory requirement of the assembler by a factor of *c*. We used ntCard (Mohamadi et al. 2017) to estimate the approximate number of singleton *k*-mers in the data set. As we describe below, an additional tracking Bloom filter is used to record *k*-mers that have been included in previously assembled contigs, and so the total memory multiplier is *c* + 1.

We now proceed to describe the unique aspects of the ABySS 2.0 algorithm in comparison to Minia. The first difference is the method used to seed graph traversals in order to generate contigs. While Minia identifies and stores branching points of the de Bruijn graph to use as starting points for contig traversal, ABySS 2.0 instead extends *solid reads* left and right until either a dead end or a branching point is encountered in the graph. A read is considered to be a solid read if it consists entirely of solid *k*-mers, and is thus likely to represent a correct path in the de Bruijn graph. The percentage of solid reads in the data set depends on the user-specified minimum *k*-mer occurrence threshold. In the case of the Genome in a Bottle HG004 assembly, the *k*-mer occurrence threshold was set to three and the number of solid reads was 782,886,725 of 868,593,056 (90.1%), after correction with BFC (Li 2015a). The read extension approach to contig generation has the advantage of being simple to implement but requires some precautions to ensure that redundant contigs are not generated by solid reads located in the same neighborhood of the de Bruijn graph. We address this issue by using an additional tracking Bloom filter to record the set of *k*-mers that have previously been included in contigs; if all the *k*-mers of a solid read are already contained in the tracking Bloom filter, it is not extended into a contig but is instead skipped. We note that in order for this scheme to work correctly, solid reads that span branching points of the de Bruijn graph must be split at the branching points and treated as separate candidates for extension. We note that the tracking Bloom filter is assigned the same size in memory as the chained Bloom filters that make up the cascading Bloom filter, described in the previous paragraph.

A second important difference between Minia and ABySS 2.0 is the strategy used for handling of Bloom filter false positives. While the Minia approach uses an additional nonprobabilistic data structure to store *critical false positives* (Chikhi and Rizk 2013), ABySS 2.0 instead uses a look-ahead mechanism during graph traversal to eliminate short branches that are caused by false positives and recurrent sequencing errors (Fig. 1C). The majority of branches created by sequencing errors are removed by the cascading Bloom filter. In detail, we invoke a look-ahead step at each branching point we encounter during contig extension, up to a distance of *k* nodes. If the look-ahead step reveals that a branch is less than or equal to *k* nodes in length, it is considered to be a false branch, and its existence is ignored. If, on the other hand, the branch point has two or more branches that are longer than *k* nodes, then the unitig extension is halted. The use of look-ahead incurs an additional computational cost to the graph traversal but obviates the requirement for additional data structures to track false positives and error *k*-mers.

A third difference between Minia and ABySS 2.0 is the use of a specialized hash function called ntHash in ABySS 2.0. The ntHash algorithm is an efficient method for computing the hash values of all consecutive *k*-mers in a DNA sequence recursively, in which the hash value for each *k*-mer is derived from the hash value of the previous *k*-mer. More specifically, ntHash is an adapted version of cyclic polynomial hashing and is used to compute normal or canonical hash values for all *k*-mers in a DNA sequence. A further feature of ntHash is fast computation of multiple hash values for the same *k*-mer, without repeating the entire hashing computation. This is a useful feature for bioinformatics applications such as ABySS 2.0 that employ a Bloom filter data structure.

### Experimental sequencing data

In our experiment to assess the effects of Bloom filter FPR on ABySS 2.0 assemblies, we used *C. elegans* N2 strain data set SRA DRR008444, consisting of Illumina GAIIx 2 × 100 bp reads on 300-bp fragments with 75-fold coverage.

For the assembler comparison, we used the data for the Ashkenazi mother (NIST HG004, Coriell cell line NA24143) from the Genome in a Bottle project (Zook et al. 2016). The Illumina WGS 2 × 250 bp paired-end sequencing data may be downloaded from the URLs listed at http://bit.ly/hg004-2x250 (SRA SRR3440461–SRR3440495). The Illumina 6-kbp mate-pair sequencing data may be downloaded from URLs listed at http://bit.ly/hg004-6kb (SRA SRR2832452–SRR283245). The BioNano optical map EXP_REFINEFINAL1_q.cmap may be downloaded from the URLs listed at http://bit.ly/hg004-bionano, and the 10x Genomics Chromium data may be downloaded from the URLs listed at http://bit.ly/hg004-chromium.

We corrected sequencing errors in the reads using the tool BFC (Li 2015a) with the parameter -s3G. We constructed the hash table of trusted *k*-mers using the paired-end reads and used this hash table to correct both the paired-end and mate-pair reads. We assembled both the BFC and uncorrected reads with each assembler (Supplemental Fig. S6; Supplemental Tables S11, S12).

We removed adapters from the mate-pair reads using NxTrim 0.4.0 (O'Connell et al. 2015) with parameters --norc --joinreads --preserve-mp. The tool also classifies the reads as mate-pair, paired-end, single-end, or unknown. We discarded the reads classified as either paired-end or single-end and, for scaffolding, used the reads classified as mate-pair and unknown, which are composed primarily of mate-pair reads originating from large fragments.

### Assembler comparison

We assembled the GIAB HG004 data set using ABySS 1.9.0 (Simpson et al. 2009), ABySS 2.0, ALLPATHS-LG 52488 (Gnerre et al. 2010), BCALM 2.0.0 (Chikhi et al. 2016), DISCOVAR de novo 52488, MaSuRCA 3.1.3 (Zimin et al. 2013), MEGAHIT 1.0.6-3-gfb1e59b (Li et al. 2016), Minia 3.0.0-alpha1 (Chikhi and Rizk 2013), SGA 0.10.14 (Simpson and Durbin 2011), and SOAPdenovo 2.04 (Luo et al. 2012). We assembled with each tool the paired-end reads corrected by BFC 181. The mate-pair reads categorized by NxTrim 0.4.0 and corrected by BFC were used for scaffolding, when applicable for that assembler. We scaffolded the DISCOVAR de novo assembly using BESST 2.2.4 (Sahlin et al. 2016), LINKS 1.8.2 (Warren et al. 2015), and ABySS-Scaffold 1.9.0 (data not shown).

Most software used in these analyses was installed from the Homebrew-Science software collection using Linuxbrew (http://linuxbrew.sh) with the command brew install abyss allpaths-lg bcalm bfc bwa discovardenovo masurca megahit nxtrim samtools seqtk sga soapdenovo. The development version of ABySS 2.0 used in the comparison was compiled from the bloom-abyss-preview tag at https://github.com/bcgsc/abyss/tree/bloom-abyss-preview, and we provide the source code in Supplemental Archive 1. Minia 3.0.0-alpha1 and LINKS 1.8.2 were installed manually, as these versions are not yet available in Linuxbrew as of this writing. The Python package besst was installed using pip install besst.

We provide the commands and configuration files used to run the various assemblers and scaffolding tools in Supplemental Listings S1 through S16 and as Makefile scripts in the Supplemental Archive 2. The scripts are also available online at https://github.com/bcgsc/abyss-2.0-giab. To calculate a suitable Bloom filter size for ABySS 2.0, we counted distinct *k*-mers in the reads with ntHash (Mohamadi et al. 2017) and targeted a Bloom filter FPR of 5%; we provide further details in “Assembler Scripts and Configuration Files” in the Supplemental Material. To assess the correctness of each assembly, we aligned the contigs to the primary chromosome sequences of human reference GRCh38 with BWA-MEM 0.7.13 and counted the number of resulting breakpoints with abyss-samtobreak -G3088269832 -l500.

## Data access

The FASTA files for the assemblies of the HG004 Genome in a Bottle data from this study may be downloaded from NCBI at http://bit.ly/ncbi-giab-abyss2 and are also mirrored at http://bit.ly/abyss2-ftp.

## Supplementary Material

Supplemental Material
