# Supplementary material for: ABySS 2.0: resource-efficient assembly of large genomes using a Bloom filter
Source: Genome Res. 2017 May;27(5):768–77. doi: 10.1101/gr.214346.116 (PMC5411771; doi:10.1101/gr.214346.116)
Supplement: Supplemental Material [file supp_27_5_768__index.html]

ABySS 2.0: resource-efficient assembly of large genomes using a Bloom filter — ABySS 2.0: resource-efficient assembly of large genomes using a Bloom filter — Supplemental Material 

# ABySS 2.0: resource-efficient assembly of large genomes using a Bloom filter

## Supplemental Material

- Supplemental\_Archive\_1.tar.gz
- Supplemental\_Archive\_2.tar.gz
- Supplemental\_Material.pdf
