# Supplementary material for: ABySS 2.0: resource-efficient assembly of large genomes using a Bloom filter
Source: Genome Res. 2017 May;27(5):768–77. doi: 10.1101/gr.214346.116 (PMC5411771; doi:10.1101/gr.214346.116)
Supplement: Supplemental Material [file supp_gr.214346.116_Supplemental_Material.pdf]

Supplemental Material of ABySS 2.0: Resource-Efficient Assembly of Large Genomes using a Bloom Filter

Shaun D Jackman Benjamin P Vandervalk
Hamid Mohamadi Justin Chu Sarah Yeo
S Austin Hammond Golnaz Jahesh Hamza Khan
Lauren Coombe Rene L Warren Inanc Birol

Contents

Effect of Bloom Filter False Positive Rate 2

Assembler Comparison Details 8
- Sealer Gap Filling Results . . . 8
- Sequence Identity and Genome Coverage . . . 8
- K-mer Size Sweeps . . . 9
- Additional Benchmarking of ABySS . . . 13
- Assemblies with Raw and BFC-corrected Reads . . . 14
- Software . . . 24
- Assembler Scripts and Configuration Files . . . 24

Shortened URLs from the Main Text 26

Listings

S1 ABySS 1.0 . . . 25
S2 ABySS 2.0. Note that the parameter `c` of the prerelease version tagged `bloom-abyss-preview` evaluated in this paper has been renamed to `kc` in the final released version of ABySS 2.0. . . . 26
S3 ALLPATHS-LG . . . 26
S4 ALLPATHS-LG `in_libs.csv` https://github.com/bcgsc/abyss-2.0-giab/blob/1.0/allpaths-lg/in_libs.csv . . . 28
S5 ALLPATHS-LG `in_groups.csv` https://github.com/bcgsc/abyss-2.0-giab/blob/1.0/allpaths-lg/in_groups.csv . . . 28
S6 BCALM 2. The largest value of k supported by BCALM 2 is 63. 28

S7 DISCOVAR *de novo*. Assemble the paired-end reads using DISCOVAR *de novo* and scaffold with ABySS-Scaffold, BESST and LINKS. . . . 29
S8 MaSuRCA. The script `assemble.sh` is generated by `masurca` itself. 29
S9 MaSuRCA `config.txt` https://github.com/bcgsc/abyss-2.0-giab/blob/1.0/masurca/config.txt . . . 29
S10 MEGAHIT. The variable `$pe400` is the list of paths to the BFC-corrected, interleaved, and gzipped paired-end FASTQ files. . . . 29
S11 Minia. The largest value of k supported by Minia is 128. . . . 30
S12 SGA . . . 30
S13 SOAPdenovo2 . . . 30
S14 SOAPdenovo2 `hsapiens.config` https://github.com/bcgsc/abyss-2.0-giab/blob/1.0/soapdenovo/hsapiens.config . . . 31
S15 BioNano `hybridScaffold.pl` . . . 31
S16 ARCS. The script `makeTSVfile.py` is available online at https://github.com/sarahyeo/giab. . . . 31

Effect of Bloom Filter False Positive Rate

To assess the effects of the Bloom filter false positive rate (FPR) on ABySS 2.0 assemblies, we conducted assemblies of the *C. elegans* N2 strain DRR008444 dataset (Illumina GA IIx sequencing of 2x100 bp reads of 300 bp fragments to 75 fold coverage) under a range of different Bloom FPR values and assessed the resulting NG50, number of misassemblies, wallclock times. We note that Bloom filter false positive rate is determined by a combination of Bloom filter size, number of Bloom filter hash functions, and number of distinct k-mers in the dataset, as per Equation 1 in the main text. However, the prediction of Bloom filter FPR is further complicated by the use of a cascading chain of Bloom filters to remove low-occurrence k-mers, as detailed in the assembly algorithm description in Methods. For the purposes of our experiment, we fixed all parameters affecting FPR except the Bloom filter memory allocation, which was used as the driving parameter for the experiment. In particular, we fixed the number of Bloom filter hash functions at 1, fixed the number of cascading Bloom filters at 4, fixed the k-mer size at 64, and varied the Bloom filter memory allocation from 250 MB to 3000 MB with a step size of 250 MB. For example, the ABySS 2.0 assembly for a Bloom filter memory allocation of 250 MB was run with the command `abyss-pe c=4 k=64 H=1 B=250M in='DRR008444_1.fastq DRR008444_2.fastq'`, where `c=4` specifies the use of 4 cascading Bloom filters (i.e. minimum k-mer count threshold of 4), `k=64` specifies a k-mer size of 64, and `H=1` specifies that the Bloom filter should use a single hash function. The runs for other Bloom filter sizes used the same parameter values with the exception of `B` (Bloom filter memory allocation). The wallclock time of the assemblies was measured with `/usr/bin/time` and the false positive rates corresponding to each Bloom filter size were obtained from the ABySS 2.0 log files. All assemblies

were run with 12 threads on an isolated machine with 48GB RAM and two Xeon X5650 CPUs.

We used QUAST 3.2 to calculate the NG50 and misassembly metrics for the experiment, using the *C. elegans* Bristol N2 strain as the reference genome (NCBI BioProject PRJNA158). Expanding on the results presented in Fig. 3 of the main text, Fig. S1, S2, S3 depict the changes to FPR, wallclock time, and a variety of QUAST contiguity and misassembly metrics that result from changing the Bloom filter allocation, while Tables S1, S2, S3 provide the corresponding data. We observe that as the Bloom filter memory decreases from 3000 MB to 500 MB (FPR values of 1.91% and 10.9%, respectively), the majority of assembly metrics remain stable. However, large changes in the metrics occur when the Bloom filter allocation is decreased further from 500 MB to 250 MB (FPR values of 10.9% and 20.7%, respectively). We similarly observe a steep increase in wallclock time from 57 min to 152 min when further decreasing Bloom filter allocation from 500 MB to 250 MB. These results indicate that a target FPR between 5% - 10% provides the best trade-off between assembly quality, wallclock time, and memory usage.

Table S1: Bloom filter memory, N50, NG50, NGA50, number of misassemblies, number of relocation misassemblies, and number of misassembled contigs reported by QUAST 3.2 for ABySS 2.0 assemblies of *C. elegans* dataset DRR008444, using the C. elegans Bristol N2 strain as the reference genome (NCBI BioProject PRJNA158). Results are shown for Bloom filter memory allocations ranging between 250 MB and 3000 MB with a step size 250 MB. Number of translocation misassemblies and inversion misassemblies are omitted because their count was zero across all Bloom filter sizes.

Bloom Filter Mem (MB)	N50	NG50	NGA50	Misassemblies	Relocations	Misassembled Contigs
250	10512	9306	9294	9	9	9
500	10954	9629	9608	9	9	9
750	10973	9610	9607	9	9	9
1000	10956	9607	9603	9	9	9
1250	10984	9607	9603	9	9	9
1500	10987	9608	9606	9	9	9
1750	10980	9607	9598	9	9	9
2000	10970	9607	9597	9	9	9
2250	10973	9594	9587	9	9	9
2500	10973	9587	9584	9	9	9
2750	10970	9591	9586	9	9	9
3000	10970	9597	9594	9	9	9

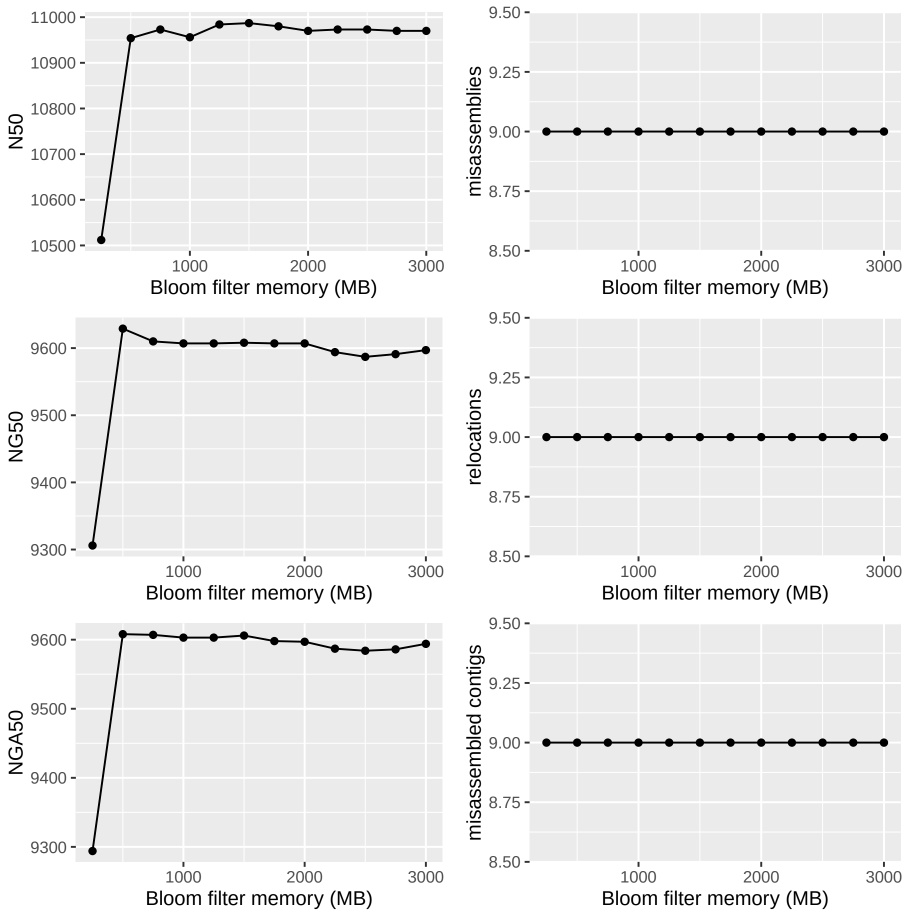

Figure S1: N50, number of misassemblies, NG50, number of relocation misassemblies, NGA50, and number of misassembled contigs reported by QUAST 3.2 for ABySS 2.0 assemblies of *C. elegans* dataset DRR008444, using the C. elegans Bristol N2 strain as the reference genome (NCBI BioProject PRJNA158). Results are shown for Bloom filter memory allocations ranging between 250 MB and 3000 MB with a step size 250 MB. Number of translocation misassemblies and inversion misassemblies are omitted because their count was zero across all Bloom filter sizes.

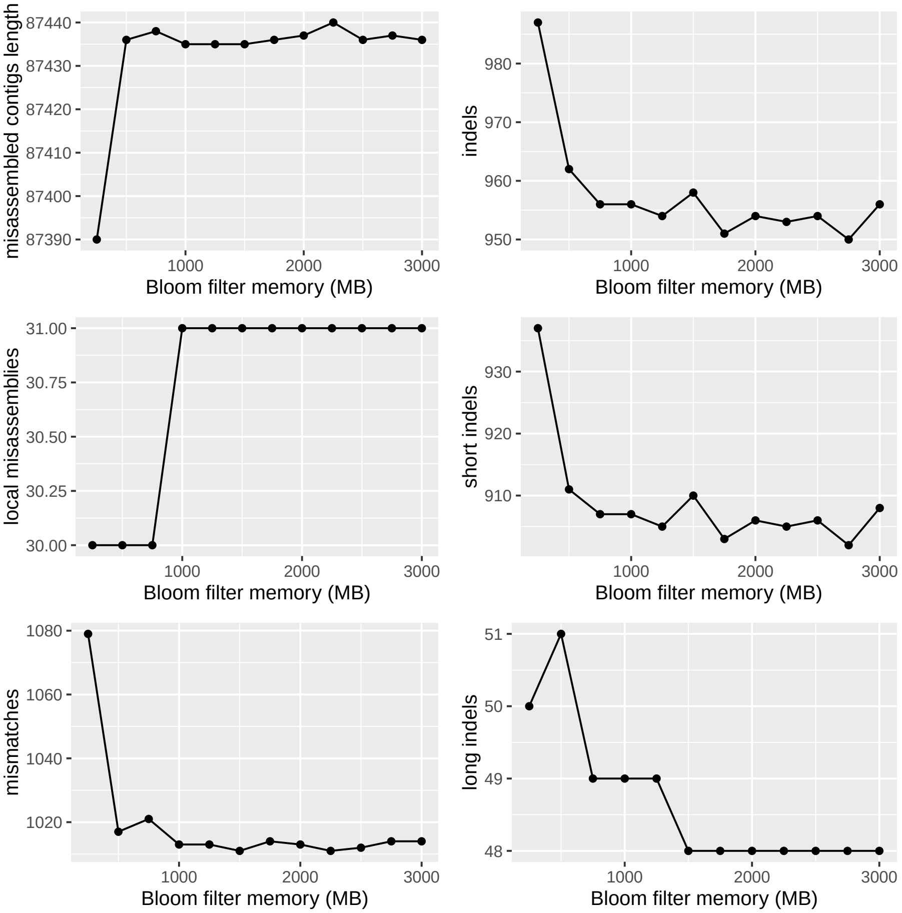

Figure S2: Sum length of misassembled contigs, number of indels, number of local misassemblies, number of short indels, number of mismatches, and number of long indels reported by QUAST 3.2 for ABySS 2.0 assemblies of *C. elegans* dataset DRR008444, using the C. elegans Bristol N2 strain as the reference genome (NCBI BioProject PRJNA158). Results are shown for Bloom filter allocations ranging between 250 MB and 3000 MB with a step size 250 MB.

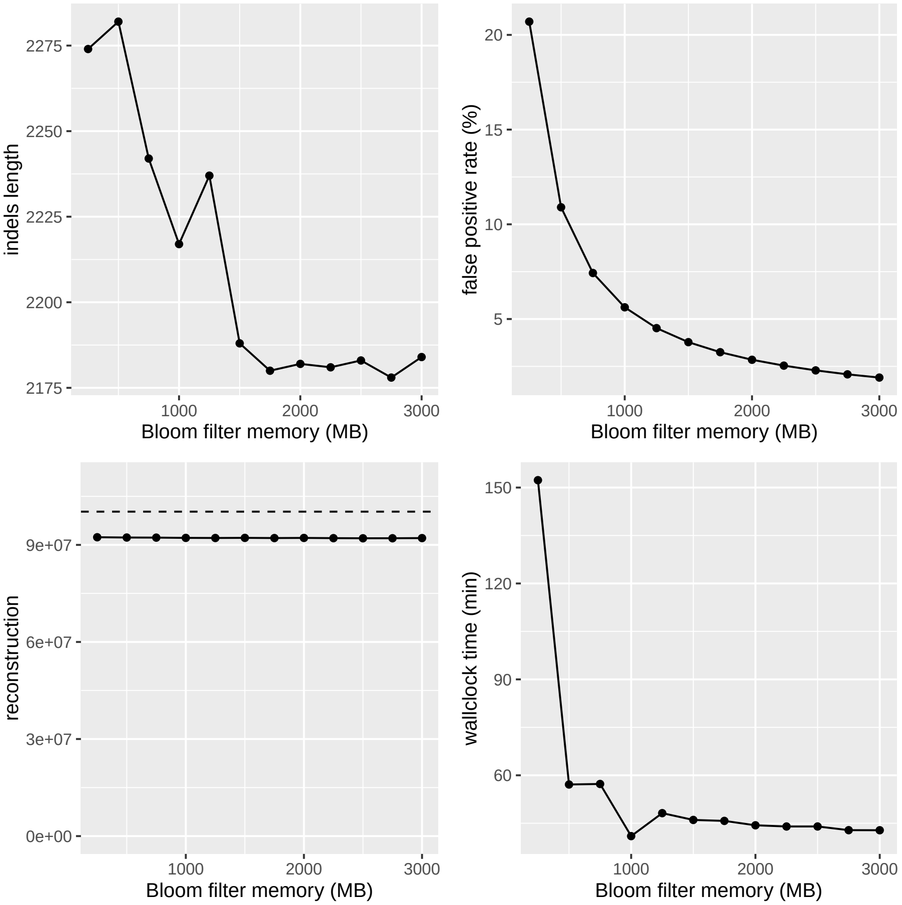

Figure S3: Sum length of indels, Bloom filter false positive rate, reconstruction, and wallclock time for ABySS 2.0 assemblies of *C. elegans* dataset DRR008444, using Bloom filter memory allocations ranging between 250 MB and 3000 MB with a step size 250 MB. Sum length of indels and reconstruction were computed by QUAST 3.2 using the C. elegans Bristol N2 strain as the reference genome (NCBI BioProject PRJNA158). The reconstruction figure corresponds to the "Total_length" column reported by QUAST, which the sum length of all assembled sequences >= 500 bp. The dashed line of the reconstruction plot indicates the length of the reference genome sequence.

Table S2: Bloom filter memory, sum length of misassembled contigs, number of local misassemblies, number of mismatches, number of indels, number of short indels, and number of long indels reported by QUAST 3.2 for ABySS 2.0 assemblies of *C. elegans* dataset DRR008444, using the C. elegans Bristol N2 strain as the reference genome (NCBI BioProject PRJNA158). Results are shown for Bloom filter memory allocations ranging between 250 MB and 3000 MB with a step size 250 MB.

Bloom Filter Mem (MB)	Misassembled Contigs Length	Local Misassemblies	Mismatches	Indels	Short Indels	Long Indels
250	87390	30	1079	987	937	50
500	87436	30	1017	962	911	51
750	87438	30	1021	956	907	49
1000	87435	31	1013	956	907	49
1250	87435	31	1013	954	905	49
1500	87435	31	1011	958	910	48
1750	87436	31	1014	951	903	48
2000	87437	31	1013	954	906	48
2250	87440	31	1011	953	905	48
2500	87436	31	1012	954	906	48
2750	87437	31	1014	950	902	48
3000	87436	31	1014	956	908	48

Table S3: Bloom filter memory, sum length of indels, reconstruction, Bloom filter false positive rate, and wallclock time for ABySS 2.0 assemblies of *C. elegans* dataset DRR008444, using Bloom filter memory allocations ranging between 250 MB and 3000 MB with a step size 250 MB. Sum length of indels and reconstruction were computed by QUAST 3.2 using the C. elegans Bristol N2 strain as the reference genome (NCBI BioProject PRJNA158). The reconstruction figure corresponds to the "Total_length" column reported by QUAST, which the sum length of all assembled sequences >= 500 bp. The dashed line of the reconstruction plot indicates the length of the reference genome sequence.

Bloom Filter Mem (MB)	Indels Length	Reconstruction	False Positive Rate	Wallclock (min)
250	2274	92365990	20.7	152.31
500	2282	92276322	10.9	57.13
750	2242	92248132	7.43	57.29
1000	2217	92162266	5.62	40.98
1250	2237	92135097	4.52	48.17
1500	2188	92166918	3.78	46.04
1750	2180	92112941	3.25	45.74

Bloom Filter Mem (MB)	Indels Length	Reconstruction	False Positive Rate	Wallclock (min)
2000	2182	92152839	2.85	44.37
2250	2181	92081875	2.54	43.99
2500	2183	92045926	2.29	44.00
2750	2178	92055208	2.08	42.84
3000	2184	92099533	1.91	42.82

Assembler Comparison Details

Sealer Gap Filling Results

In addition to comparing the contiguity and correctness of the contig sequences in Fig. 3A, we also assessed the contiguity improvements produced by closing scaffold gaps with Sealer, prior to splitting the sequences at 'N's. Sealer is a tool that fills scaffold gaps by searching for a connecting path between gap flanks in the de Bruijn graph, using multiple k-mer sizes. For the ABySS 1.0 assembly, Sealer closed 33,380 of 148,795 (22.4%) of scaffold gaps and increased the contig NG50 from 30 kbp to 38 kbp. For the ABySS 2.0 assembly, Sealer closed 33,533 of 213,480 (15.7%) scaffolds and increased the contig NG50 from 21 kbp to 25 kbp.

Sequence Identity and Genome Coverage

We aligned the contigs to the reference genome using BWA-MEM and filtered out secondary alignments (SAM flag 0x100). We calculated the number of reference nucleotides covered by contigs and the total number of aligned contig nucleotides using `samtools depth`. We calculated the total number of mismatching nucleotides by computing the sum of the SAM `NM` tag (number of mismatches) of the alignments. The percent identity is calculated as one minus the number of mismatches divided by the total number of aligned contig nucleotides. The percent genome coverage is calculated as the number of reference genome positions covered by an aligned contig divided by the number of non-N reference nucleotides, 2,937,639,113 bp.

Table S4: Percent genome coverage, percent sequence identity and the corresponding PHRED-scaled quality value of contigs aligned to the reference genome using BWA-MEM

Assembler	Genome coverage	Identity	QV
ABySS 1.0	96.0	99.71	25.4
ABySS 2.0	96.2	99.69	25.2

Assembler	Genome coverage	Identity	QV
BCALM 2	97.1	99.75	26.1
DISCOVAR *de novo*	96.7	99.64	24.4
MEGAHIT	97.6	99.54	23.4
Minia	93.6	99.78	26.7
SGA	97.2	99.50	23.0
SOAPdenovo2	93.0	99.80	27.1

K-mer Size Sweeps

For most of the assemblers, we conducted assemblies across a range of *k*-mer sizes and selected the optimal *k*-mer size based on the trade-off between maximizing contiguity (NGA50/NG50) and minimizing the number of breakpoints when aligning the sequences to the reference genome GRCh38 (Fig. S5, Tables S5, S6, S7, S8, S9). We note that Minia did not support *k*-mer sizes greater than 128 and BCALM 2 did not support *k*-mers sizes larger than 63. Assemblers for which we did not perform *k*-mer size optimization were DISCOVAR *de novo*, MEGAHIT, and SGA. In the case of DISCOVAR *de novo*, the software determines a suitable *k*-mer size automatically from the input data (author communication). In the case of MEGAHIT, the algorithm assembles across multiple *k*-mer sizes simultaneously. To better cover the full read length of 250bp, we extended the default range of *k*-mer sizes for MEGAHIT from 21,41,61,81,99 to 17,45,73,101,129,157,185,213,241 to achieve improved contiguity (NG50 of 8293 bp vs. 4058 bp) at the expense of additional running time (25.6 hours vs. 15.5 hours) and no significant increase in memory usage (196.9 GB vs. 194.5 GB). In the case of SGA, the assembly follows the string graph paradigm (Myers 2005) which accomodates variable-size overlaps, and so the *k*-mer size optimization was not needed.

Table S5: Scaffold contiguity and number of breakpoints for ABySS 1.0 assemblies of the GIAB HG004 dataset, conducted across a range of *k*-mer sizes. NGA50 and number of breakpoints were calculated by aligning the sequences to GRCh38 using BWA-MEM.

K-mer Size	Scaffold NGA50 (Mbp)	Scaffold NG50 (Mbp)	Breakpoints
96	2.77	2.79	2736
128	3.83	4.01	2966
136	4.02	4.32	2911
140	3.90	4.36	2922
144	4.36	4.61	2994
148	4.06	4.50	3050
152	4.02	4.37	3077
160	3.59	4.22	3137

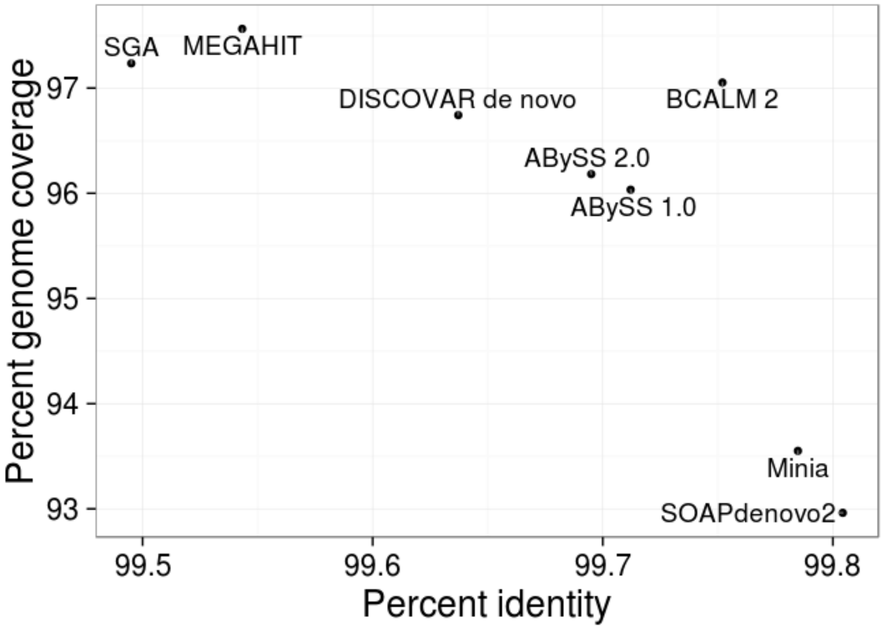

Figure S4: Percent genome coverage and percent sequence identity of contigs aligned to the reference genome using BWA-MEM

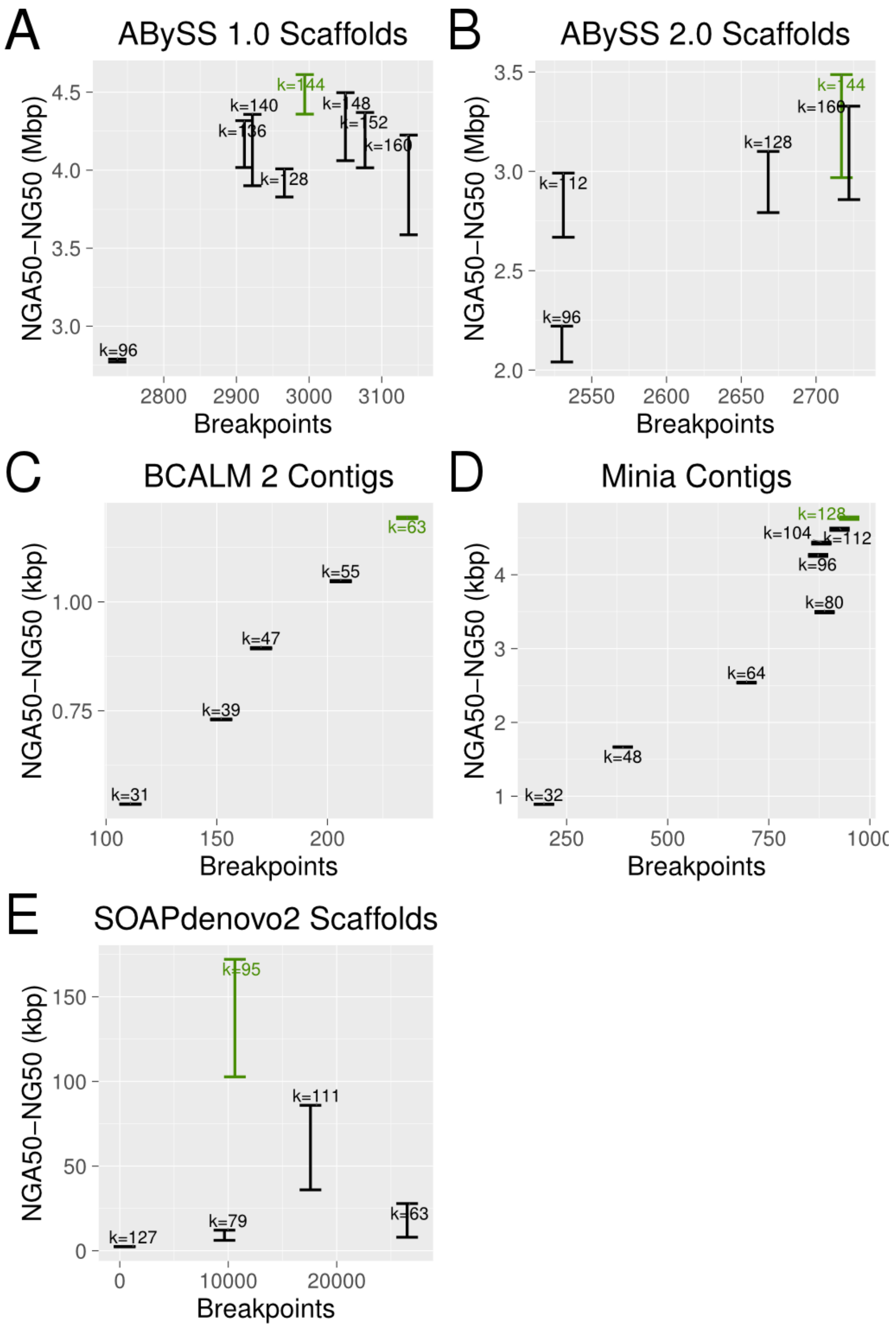

Figure S5: De Bruijn graph assembly results across a range of k-mer sizes for the Genome in a Bottle HG004 data set, using ABySS 1.0 (Panel A), ABySS 2.0 (Panel B), BCALM 2 (Panel C), Minia (Panel D), and SOAPdenovo2 (Panel E). The Y axis shows the range of NGA50 to NG50 to indicate the uncertainty caused by real genomic variants between individual HG004 and the reference genome (GRCh38), and the X axis shows the number of breakpoints that occurred when aligning the sequences to the reference genome. For each assembler, a single "best" assembly (show in green) was selected for inclusion in the assembler comparison of Fig. 3, based on the trade-off of maximizing contiguity (NGA50-NG50 range) and minimizing breakpoints.

Table S6: Scaffold contiguity and number of breakpoints for ABySS 2.0 assemblies of the GIAB HG004 dataset, conducted across a range of k-mer sizes. NGA50 and number of breakpoints were calculated by aligning the sequences to GRCh38 using BWA-MEM.

K-mer Size	Scaffold NGA50 (Mbp)	Scaffold NG50 (Mbp)	Breakpoints
96	2.04	2.22	2530
112	2.67	2.99	2531
128	2.79	3.10	2668
144	2.97	3.49	2717
160	2.86	3.33	2722

Table S7: Scaffold contiguity and number of breakpoints for BCALM 2 assemblies of the GIAB HG004 dataset, conducted across a range of k-mer sizes. NGA50 and number of breakpoints were calculated by aligning the sequences to GRCh38 using BWA-MEM.

K-mer Size	Contig NGA50 (kbp)	Contig NG50 (kbp)	Breakpoints
31	0.54	0.54	111
39	0.73	0.73	152
47	0.89	0.90	170
55	1.05	1.05	206
63	1.19	1.20	236

Table S8: Scaffold contiguity and number of breakpoints for Minia assemblies of the GIAB HG004 dataset, conducted across a range of k-mer sizes. NGA50 and number of breakpoints were calculated by aligning the sequences to GRCh38 using BWA-MEM.

K-mer Size	Contig NGA50 (kbp)	Contig NG50 (kbp)	Breakpoints
32	0.89	0.89	194
48	1.66	1.67	389
64	2.53	2.54	695
80	3.48	3.50	888
96	4.25	4.28	872
104	4.42	4.44	880
112	4.60	4.63	925
128	4.75	4.78	949

Table S9: Scaffold contiguity and number of breakpoints for SOAPdenovo2 assemblies of the GIAB HG004 dataset, conducted across a range of k-mer sizes. NGA50 and number of breakpoints were calculated by aligning the sequences to GRCh38 using BWA-MEM.

K-mer Size	Scaffold NGA50 (kbp)	Scaffold NG50 (kbp)	Breakpoints
63	7.98	27.87	26705
79	6.16	12.16	9978
95	102.71	172.11	11219
111	35.97	85.92	18144
127	2.38	2.54	1223

Additional Benchmarking of ABySS

All assemblies for the assembler comparison of Fig. 3 were run on servers with 4 Xeon E7-8867 v3 CPUs running @ 2.50GHz, having a total of 64 cores and 2.5 TB of RAM. In addition to the main runs on the Xeon E7 machines, we also conducted additional performance tests for ABySS 1.0 and ABySS 2.0 on alternate architectures.

To measure the performance of ABySS 1.0 in a cluster environment, we benchmarked an MPI assembly job distributed across 11 nodes, each having 48 GB RAM and 2 Xeon X5650 CPUs running at 2.67 GHz. Each cluster node provided a total of 12 CPU cores and the cluster nodes were interconnected via Infiniband. Table S10 compares the wallclock times of the distributed ABySS 1.0 job and the main ABySS 1.0 run from Fig. 3, which was run on a single 64-core Xeon E7 machine. For the sake of comparison, we set the number of MPI processes for the cluster assembly job to 64 (abyss-pe parameter "np=64"), even though 132 CPU cores were available across the 11 cluster nodes. Wallclock times in Table S10 are broken down by ABySS assembly stage. We note that only the first (unitig) stage of the ABySS assembly pipeline is distributed across nodes with MPI, whereas the contig and scaffold stages are multithreaded and run on a single node. As a result, the contig stage ran much more slowly for the cluster-based ABySS 1.0 job than for the single-machine Xeon E7 run (14.0 hours vs. 3.3 hours). The scaffold stage, which is not as computationally intensive as the contig stage, ran in roughly the same wallclock time in both cases (4.5 hours vs. 4.8 hours). The overall wallclock time for the distributed ABySS 1.0 assembly was 25.4 hours vs. 14.3 hours for the E7 run. In practice, the distributed ABySS 1.0 job also required more memory than the single-machine E7 run. While the E7 run had a peak memory requirement of 418 GB RAM, the cluster job required 528 GB of aggregate RAM (11 nodes with 48 GB per node). Although the actual memory used by ABySS 1.0 was the same in both cases, the cluster job required extra headroom because the distribution of k-mer data was not perfectly even across MPI processes.

Table S10: Breakdown of wallclock time for two ABySS 1.0 assemblies of the Genome in a Bottle HG004 data set, run on different platforms. The assembly corresponding to the "HPC cluster" column was an MPI job distributed across 11 cluster nodes with 48 GB RAM and 2 Xeon X5650 CPUs each. Each cluster node provided a total of 12 CPU cores. The assembly for the "Xeon E7" job was run on a single machine having 2.5 TB RAM and 4 Xeon E7-8867 v3 CPUs providing a total of 64 cores.

assembly stage	wallclock hours (HPC cluster)	wallclock hours (Xeon E7)
unitigs	6.9 (11 nodes, 64 MPI processes)	6.2 (1 node, 64 MPI processes)
contigs	14.0 (1 node, 12 threads)	3.3 (1 node, 64 threads)
scaffolds	4.5 (1 node, 12 threads)	4.8 (1 node, 64 threads)

To test the performance of ABySS 2.0 on a low-memory machine, we benchmarked the ABySS 2.0 on a node with 48 GB RAM and 2 Xeon X5650 CPUs running at 2.67 GHz, having a total of 12 cores. As expected, the peak RAM usage was the same as for the E7 run (34 GB), while the wallclock time was approximately 4 times longer (80 hours vs. 20 hours). We attribute the longer wallclock time to the use of 12 threads rather than 64 threads, due to the lower number of cores available on this machine in comparison to the Xeon E7 server.

Assemblies with Raw and BFC-corrected Reads

To assess the impact of using BFC-corrected reads in our assembly comparison of Fig. 3, we ran equivalent assemblies on the uncorrected reads and compared the contig NGA50, contig NG50, alignment breakpoints, peak memory usage, and wallclock time to the values measured for BFC-corrected reads (Fig. S6, Tables S11, S12). For each assembler, we used identical command line parameters for the uncorrected reads assembly as were used on the BFC-corrected reads (see Methods). We note that the BFC-corrected assemblies generally required less time and memory and produced improved assembly contiguity in comparison to the uncorrected reads. Two minor exceptions were: (i) DISCOVAR *de novo* whose contig NG50 and NGA50 were 1.9% and 2.0% less respectively with the BFC corrected reads, and (ii) SGA which ran slightly faster on uncorrected reads (60 hours vs. 65 hours). For consistency, we used the assemblies of BFC corrected reads for all assemblers in Fig. 3 of the main text.

Table S11: The sequence contiguity and number of breakpoints of assemblies using raw and BFC-corrected reads from the GIAB HG004 dataset. NGA50 and number of breakpoints were calculated by aligning the sequences to GRCh38 using BWA-MEM.

Assembly	Reads	Contig NG50 (kbp)	Contig NGA50 (kbp)	Breakpoints
ABySS 1.0	BFC	30.0	29.1	1898

Assembly	Reads	Contig NG50 (kbp)	Contig NGA50 (kbp)	Breakpoints
ABySS 1.0 + Sealer	BFC	38.2	36.3	2268
ABySS 1.0	raw	19.9	19.5	1797
ABySS 1.0 + Sealer	raw	27.6	26.5	2278
ABySS 2.0	BFC	20.6	20.1	1813
ABySS 2.0 + Sealer	BFC	24.5	23.7	2089
ABySS 2.0	raw	14.2	13.9	1672
ABySS 2.0 + Sealer	raw	17.1	16.6	2153
BCALM 2	BFC	1.2	1.2	236
BCALM 2	raw	1.2	1.2	239
DISCOVAR *de novo*	BFC	82.1	76.6	1947
DISCOVAR *de novo*	raw	83.7	78.2	1958
MEGAHIT	BFC	8.3	8.1	1709
MEGAHIT	raw	4.7	4.6	1700
Minia	BFC	4.8	4.8	949
Minia	raw	3.9	3.9	878
SGA	BFC	7.9	7.9	859
SGA	raw	5.8	5.7	764
SOAPdenovo2	BFC	3.8	3.7	609

Table S12: The peak memory usage and wall clock run time of assemblies using raw and BFC-corrected reads from the GIAB HG004 dataset. Each assembler was run with 64 threads.

Assembly	Reads	Memory (GB)	Time (h)
ABySS 1.0	BFC	418	14
ABySS 1.0 + Sealer	BFC	418	39
ABySS 1.0	raw	975	16
ABySS 1.0 + Sealer	raw	975	46
ABySS 2.0	BFC	34	20
ABySS 2.0 + Sealer	BFC	34	51
ABySS 2.0	raw	57	22
ABySS 2.0 + Sealer	raw	57	53
BCALM 2	BFC	5	9
BCALM 2	raw	5	9
DISCOVAR *de novo*	BFC	618	26
DISCOVAR *de novo*	raw	640	28
MEGAHIT	BFC	197	26
MEGAHIT	raw	321	33
Minia	BFC	137	20
Minia	raw	138	27
SGA	BFC	82	65
SGA	raw	89	60
SOAPdenovo2	BFC	659	35

Assembly	Reads	Memory (GB)	Time (h)

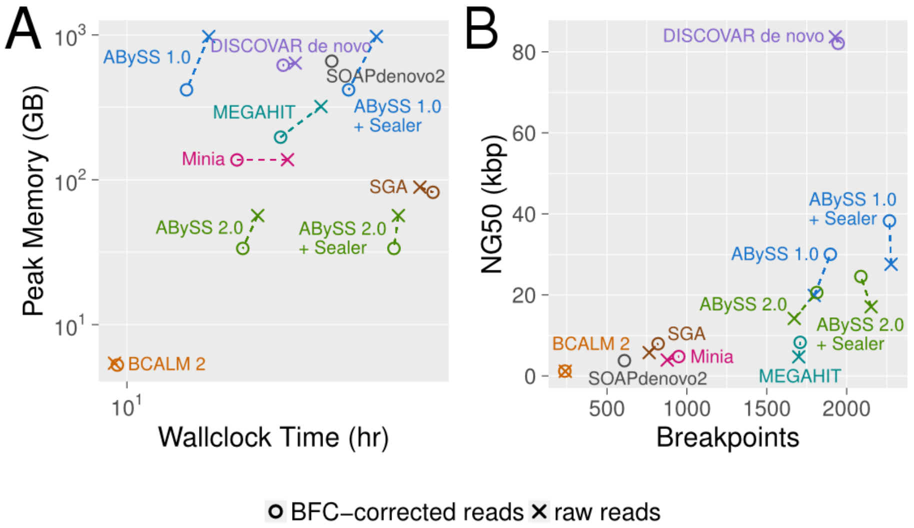

Figure S6: Comparison of assembly results for uncorrected reads and BFC-corrected reads for the GIAB HG004 dataset using ABySS 1.0, ABySS 2.0, BCALM 2, DISCOVAR *de novo*, Minia, SGA, and SOAPdenovo2. (A) Peak memory usage and wallclock times of each assembler when run on raw and BFC-corrected reads. (B) NG50 and number of breakpoints for contig sequences generated from raw and BFC-corrected reads. The number of breakpoints was calculated by aligning the assembled sequences to the reference genome GRCh38 with BWA MEM 0.7.13. For assemblies with scaffolding stages, the contigs were extracted by splitting the sequences at 'N' characters. While the SOAPdenovo2 assembly of BFC-corrected reads completed successfully, the SOAPdenovo2 assembly on uncorrected reads failed with a segmentation fault, and thus only the BFC-corrected result is shown.

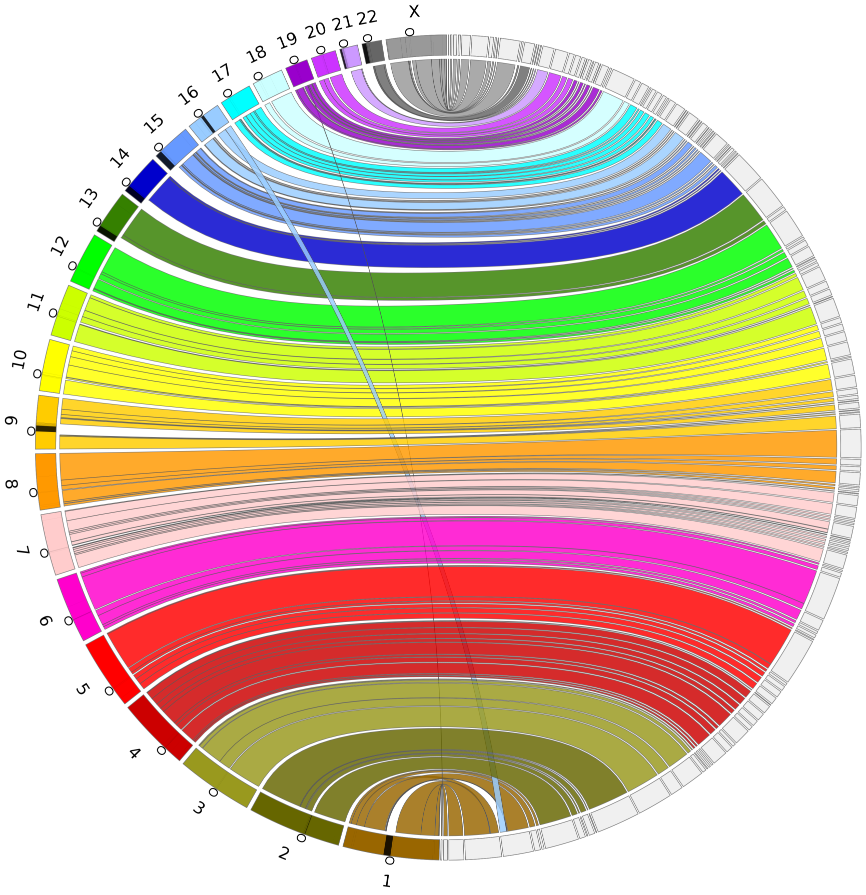

Figure S7: A Circos Assembly Consistency Plot for the ABySS 1.0 + BioNano Assembly. Scaftigs from the largest scaffolds that compose 90% of the genome are aligned to GRCh38 using BWA-MEM. GRCh38 chromosomes are displayed on the left and the scaffolds on the right. Connections show the aligned regions between the genome and scaffolds. Contigs are included as a part of the same region if the are within 1Mbp of on either side of the connection, and regions shorter than 100 kbp are not shown. The black regions on the chromosomes indicate gaps in the reference and the circles indicate the centromere location on each chromosome.

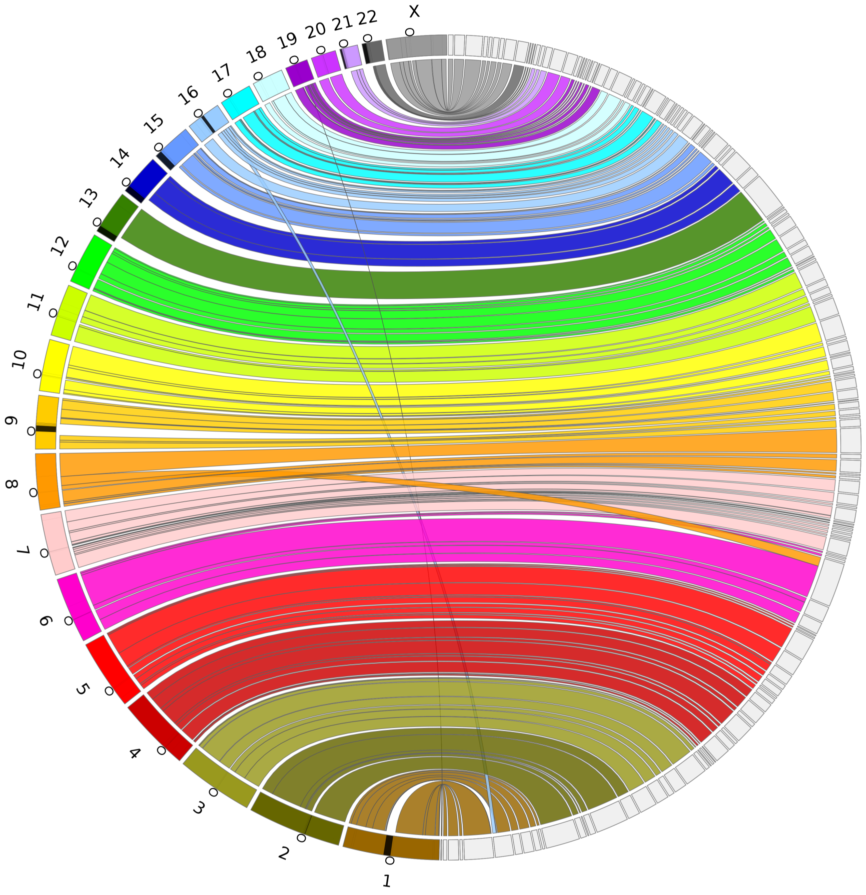

Figure S8: A Circos Assembly Consistency Plot for the ABySS 2.0 + BioNano Assembly. Scaftigs from the largest scaffolds that compose 90% of the genome are aligned to GRCh38 using BWA-MEM. GRCh38 chromosomes are displayed on the left and the scaffolds on the right. Connections show the aligned regions between the genome and scaffolds. Contigs are included as a part of the same region if the are within 1Mbp of on either side of the connection, and regions shorter than 100 kbp are not shown. The black regions on the chromosomes indicate gaps in the reference and the circles indicate the centromere location on each chromosome.

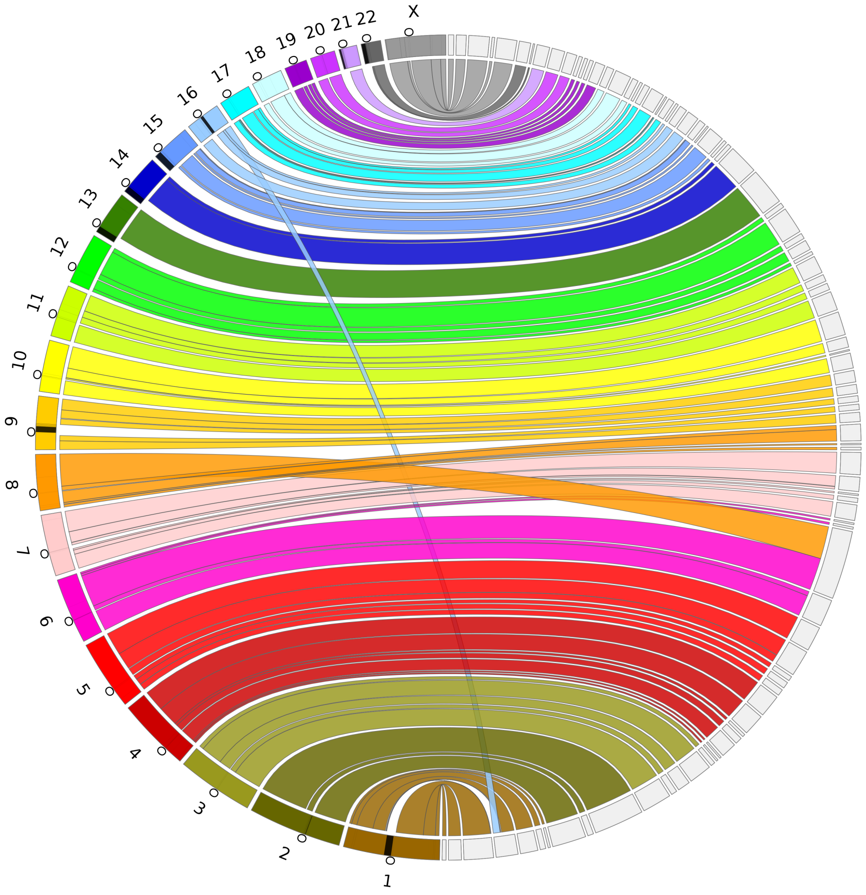

Figure S9: A Circos Assembly Consistency Plot for the ABySS 2.0 + BioNano + Chromium Assembly. Scaftigs from the largest scaffolds that compose 90% of the genome are aligned to GRCh38 using BWA-MEM. GRCh38 chromosomes are displayed on the left and the scaffolds on the right. Connections show the aligned regions between the genome and scaffolds. Contigs are included as a part of the same region if the are within 1Mbp of on either side of the connection, and regions shorter than 100 kbp are not shown. The black regions on the chromosomes indicate gaps in the reference and the circles indicate the centromere location on each chromosome.

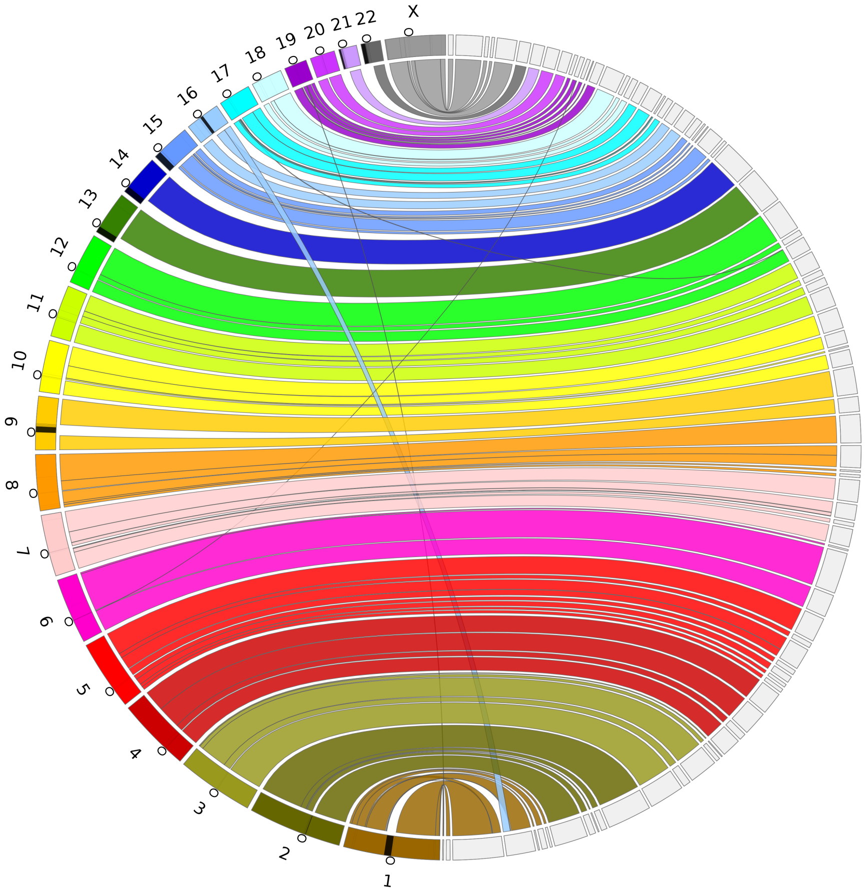

Figure S10: A Circos Assembly Consistency Plot for the DISCOVAR *de novo* + ABySS-Scaffold + BioNano Assembly. Scaftigs from the largest scaffolds that compose 90% of the genome are aligned to GRCh38 using BWA-MEM. GRCh38 chromosomes are displayed on the left and the scaffolds on the right. Connections show the aligned regions between the genome and scaffolds. Contigs are included as a part of the same region if the are within 1Mbp of on either side of the connection, and regions shorter than 100 kbp are not shown. The black regions on the chromosomes indicate gaps in the reference and the circles indicate the centromere location on each chromosome.

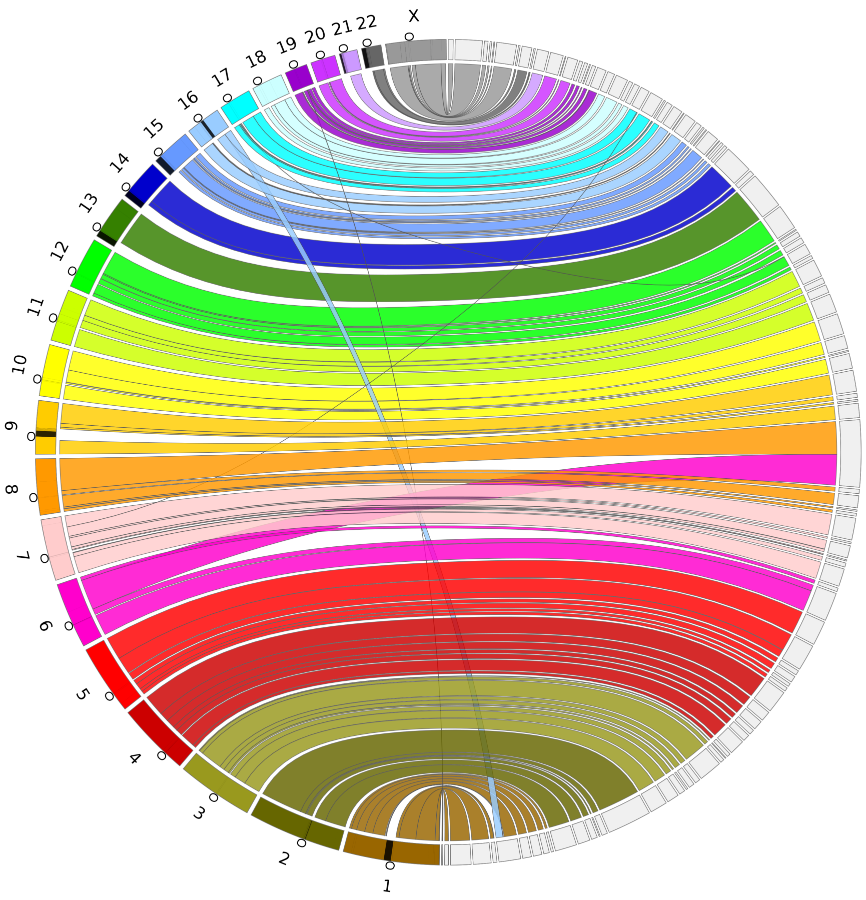

Figure S11: A Circos Assembly Consistency Plot for the DISCOVAR *de novo* + BESST + BioNano Assembly. Scaftigs from the largest scaffolds that compose 90% of the genome are aligned to GRCh38 using BWA-MEM. GRCh38 chromosomes are displayed on the left and the scaffolds on the right. Connections show the aligned regions between the genome and scaffolds. Contigs are included as a part of the same region if the are within 1Mbp of on either side of the connection, and regions shorter than 100 kbp are not shown. The black regions on the chromosomes indicate gaps in the reference and the circles indicate the centromere location on each chromosome.

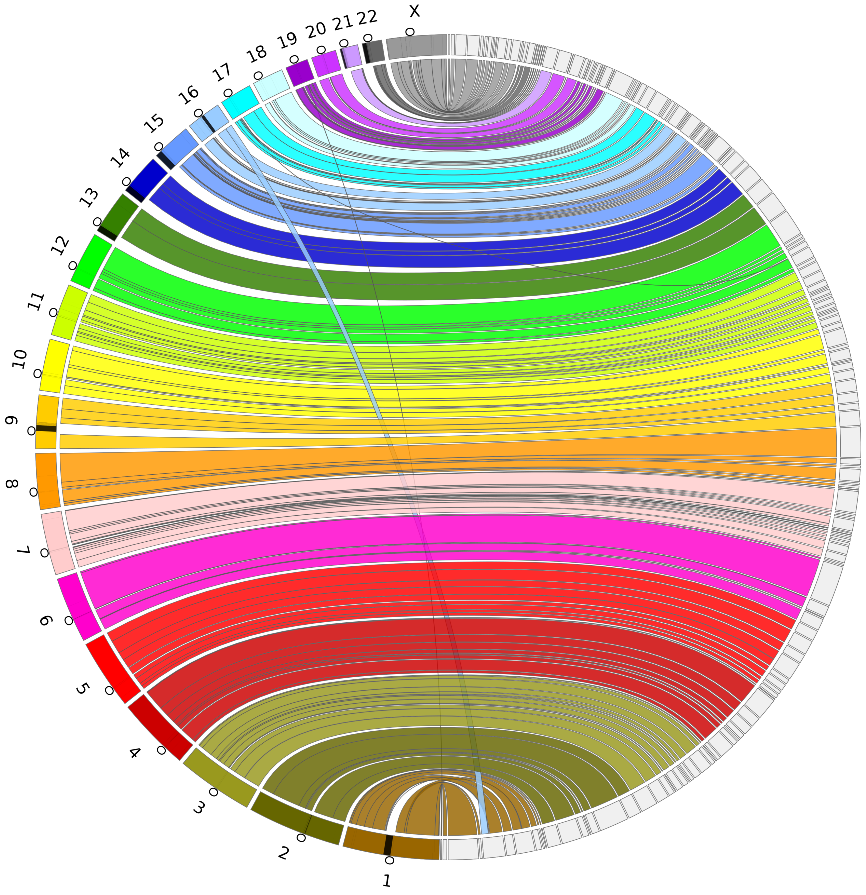

Figure S12: A Circos Assembly Consistency Plot for the DISCOVAR *de novo* + LINKS + BioNano Assembly. Scaftigs from the largest scaffolds that compose 90% of the genome are aligned to GRCh38 using BWA-MEM. GRCh38 chromosomes are displayed on the left and the scaffolds on the right. Connections show the aligned regions between the genome and scaffolds. Contigs are included as a part of the same region if the are within 1Mbp of on either side of the connection, and regions shorter than 100 kbp are not shown. The black regions on the chromosomes indicate gaps in the reference and the circles indicate the centromere location on each chromosome.

Software

Most software used in these analyses was installed from the Homebrew-Science software collection using Linuxbrew with the command `brew install abyss allpaths-lg bcalm bfc bwa discovardenovo masurca megahit nxtrim samtools seqtk sga soapdenovo`. The following three tools were installed manually.

- ABySS 2.0 preview: https://github.com/bcgsc/abyss/tree/bloom-abyss-preview
- LINKS 1.8.2: http://www.bcgsc.ca/platform/bioinfo/software/links/releases/1.8.2
- Minia 3.0.0-alpha1: https://github.com/GATB/minia/archive/v3.0.0-alpha1.tar.gz

Assembler Scripts and Configuration Files

For ABySS 1.0 (Simpson et al. 2009), we installed version 1.9.0 and assembled the paired-end and mate-pair reads with the command shown in Supplemental Listing S1, where the files `pe400.in` and `mp6k+unknown.in` are lists of the locations of compressed FASTQ files.

For ABySS 2.0, we assembled the paired-end and mate-pair reads with the command shown in Supplemental Listing S2. In comparison to the ABySS 1.0 assembly command, three Bloom filter-specific assembly parameters were added (`B=26G H=4 kc=3`), which specify the total memory allocated to the Bloom filters, the number of Bloom filter hash functions, and the number of cascading Bloom filter levels, respectively. We determined the values for total memory size (`B`) and number of hash functions (`H`) by counting distinct 144-mers with ntCard (Mohamadi et. al 2017) and targeting a false positive rate of 5% for the first level of the cascading Bloom filter. We deemed 5% to be a suitable upper bound for Bloom filter FPR based on the results of our *C. elegans* experiment above, which indicated good performance in the range of 5-10% FPR. We determined the optimal number of cascading Bloom filter levels by running assemblies with `kc=2`, `kc=3`, and `kc=4`, and choosing the assembly with highest NG50 and lowest number of breakpoints. Note that the parameter `kc` of the final release version of ABySS 2.0 was originally named `c` in the prerelease version tagged `bloom-abyss-preview` evaluated in this paper.

For ALLPATHS-LG (Gnerre et al. 2010), we installed version 52488 and attempted to assemble the paired-end and mate-pair reads with the command shown in Supplemental Listing S3 and the configuration files `in_libs.csv` and `in_groups.csv` shown in Supplemental Listings S4–S5. We terminated the ALLPATHS-LG job after it ran for more than a month without completing.

For BCALM 2 (Chikhi et al. 2016), we installed version 2.0.0 and assembled the paired-end reads with the command shown in Supplemental Listing S6. The largest value of k supported by BCALM 2 is 63.

For DISCOVAR *de novo*, the whole genome de novo assembly successor of DISCOVAR (Weisenfeld et al. 2014), we installed version 52488 and assembled the paired-end reads and scaffolded this assembly using three standalone scaffolding tools, ABySS-Scaffold 1.9.0, BESST 2.2.4 (Sahlin et al. 2016), and LINKS 1.8.2 (Warren et al. 2015), with the command shown in Supplemental Listing S7.

For MaSuRCA (Zimin et al. 2013), we installed version 3.1.3 and attempted to assemble the paired-end and mate-pair reads with the command shown in Supplemental Listing S8 and the configuration file `config.txt` shown in Supplemental Listing S9. MaSuRCA ran for five days and failed with a segmentation fault in the program `gatekeeper`.

For MEGAHIT (Li et al. 2016), we installed version 1.0.6-3-gfb1e59b and assembled the paired-end reads with the command shown in Supplemental Listing S10.

For Minia (Chikhi et al. 2013), we installed version 3.0.0-alpha1 and assembled the paired-end reads with the command shown in Supplemental Listing S11. The largest value of k supported by Minia was 128.

For SGA (Simpson and Durbin 2011), we installed version 0.10.14 and assembled the paired-end reads with the command shown in Supplemental Listing S12.

For SOAPdenovo2 (Luo et al. 2012), we installed version 2.04 and assembled the paired-end and mate-pair reads with the command shown in Supplemental Listing S13 and the configuration file `hsapiens.config` shown in Supplemental Listing S14.

We used the BioNano optical map to further scaffold the ABySS 1.0, ABySS 2.0 and DISCOVAR *de novo* assemblies, scaffolded with ABySS-Scaffold, BESST and LINKS, using IrysSolve 2.1.5063 with the command shown in Supplemental Listing S15 according to the document "Theory Of Operation: Hybrid Scaffolding" available online at http://bit.ly/bionano-scaffolding. The configuration files are used unmodified as distributed by BioNano Genomics and available online at https://github.com/bcgsc/abyss-2.0-giab/tree/master/bionano.

We used 10x Genomics Chromium data to scaffold the ABySS 2.0 + BioNano scaffolds with ARCS (Yeo et al. 2017) and LINKS 1.8.2 (Warren et al. 2015). The version of ARCS used in the paper is available from: https://github.com/bcgsc/arcs/tree/arcs-prerelease. We aligned the Chromium reads to the ABySS 2.0 + BioNano scaffolds using BWA-MEM with default settings and ran ARCS and LINKS with the commands shown in Supplemental Listing S16.

Listing S1 ABySS 1.0

```
abyss-pe name=hsapiens np=64 k=144 q=15 v=-v l=40 s=1000 n=10 \
    S=1000-10000 N=15 mp6k_de=--mean mp6k_n=1 \
    lib=pe400 pe400=$(<pe400.in) \
    mp=mp6k mp6k=$(<mp6k+unknown.in)
```

Listing S2 ABySS 2.0. Note that the parameter `c` of the prerelease version tagged `bloom-abyss-preview` evaluated in this paper has been renamed to `kc` in the final released version of ABySS 2.0.

```
abyss-pe name=hsapiens np=64 k=144 q=15 v=-v l=40 s=1000 n=10 \
    B=26G H=4 c=3 \
    S=1000-10000 N=7 mp6k_de=--mean mp6k_n=1 \
    lib=pe400 pe400=$(<pe400.in) \
    mp=mp6k mp6k=$(<mp6k+unknown.in)
```

Listing S3 ALLPATHS-LG

```
PrepareAllPathsInputs.pl DATA_DIR=$PWD PLOIDY=2 HOSTS=32
RunAllPathsLG PRE=. REFERENCE_NAME=. DATA_SUBDIR=. RUN=allpaths SUBDIR=run
```

Shortened URLs from the Main Text

Table S13: Shortened URLs

Short URL	Full URL
http://bit.ly/hiseq2500	http://www.illumina.com/systems/hiseq_2500_1500/performance_specifications.html
http://bit.ly/cornell-price-list	http://www.biotech.cornell.edu/brc/genomics/services/price-list
http://bit.ly/bionano-scaffolding	http://bionanogenomics.com/wp-content/uploads/2016/04/30073-Rev-A-Hybrid-Scaffolding-Theory-of-Operations.pdf
http://bit.ly/hg004-2x250	https://github.com/genome-in-a-bottle/giab_data_indexes/blob/master/AshkenazimTrio/sequence.index.AJtrio_Illumina_2x250bps_06012016
http://bit.ly/hg004-6kb	https://github.com/genome-in-a-bottle/giab_data_indexes/blob/master/AshkenazimTrio/sequence.index.AJtrio_Illumina_6kb_matepair_wgs_08032015
http://bit.ly/hg004-bionano	https://github.com/genome-in-a-bottle/giab_data_indexes/blob/master/AshkenazimTrio/alignment.index.AJtrio_BioNano_xmap_cmap_GRC37_10012015

Short URL	Full URL
http://bit.ly/hg004-chromium	https://github.com/genome-in-a-bottle/giab_data_indexes/blob/master/AshkenazimTrio/alignment.index.AJtrio_10Xgenomics_ChromiumGenome_GRCh37_GRCh38_06202016
http://bit.ly/ncbi-giab-abyss2	ftp://ftp-trace.ncbi.nlm.nih.gov/giab/ftp/data/AshkenazimTrio/analysis/BCGSC_HG004_ABySS2.0_assemblies_12082016/
http://bit.ly/abyss2-ftp	ftp://ftp.bcgsc.ca/supplementary/abyss2

Listing S4 ALLPATHS-LG `in_libs.csv`
https://github.com/bcgsc/abyss-2.0-giab/blob/1.0/allpaths-lg/in_libs.csv

```
library_name,project_name,organism_name,type,paired,frag_size,frag_stddev,
    insert_size,insert_stddev,read_orientation,genomic_start,genomic_end
pe400,giab,hsapiens,fragment,1,400,90,0,0,inward,0,0
mp6k,giab,hsapiens,jumping,1,0,0,6000,1400,outward,0,0
```

Listing S5 ALLPATHS-LG `in_groups.csv`
https://github.com/bcgsc/abyss-2.0-giab/blob/1.0/allpaths-lg/in_groups.csv

```
max_rd_len=250

[LIB]
avg_ins=400
asm_flags=3
rank=1
pair_num_cutoff=3
map_len=32
p=D3_S1_L001_001.bfc.fa.gz
p=D3_S1_L001_002.bfc.fa.gz
p=D3_S1_L001_003.bfc.fa.gz
p=D3_S1_L001_004.bfc.fa.gz
...
p=D3_S3_L002_001.bfc.fa.gz
p=D3_S3_L002_002.bfc.fa.gz
p=D3_S3_L002_003.bfc.fa.gz
p=D3_S3_L002_004.bfc.fa.gz

[LIB]
avg_ins=6000
reverse_seq=1
asm_flags=2
rank=2
pair_num_cutoff=5
map_len=35
p=MPHG004-23100079/MPHG004_S3_L003_001.mp.bfc.fa.gz
p=MPHG004-23110109/MPHG004_S3_L003_001.mp.bfc.fa.gz
p=MPHG004-23100079/MPHG004_S3_L003_001.unknown.bfc.fa.gz
p=MPHG004-23110109/MPHG004_S3_L003_001.unknown.bfc.fa.gz
```

Listing S6 BCALM 2. The largest value of k supported by BCALM 2 is 63.

```
bcalm -in pe400.in -out hsapiens-unitigs -k 63 -abundance 5 -nb-cores 64
bglue -in hsapiens-unitigs.h5 -out hsapiens-unitigs -k 63
```

Listing S7 DISCOVAR *de novo*. Assemble the paired-end reads using DISCOVAR *de novo* and scaffold with ABySS-Scaffold, BESST and LINKS.

```
DiscovarDeNovo MAX_MEM_GB=750 READS=@pe400.in OUT_DIR=./hsapien
abyss-pe name=hsapiens mp=mp6k j=64 k=200 l=40 s=500 S=500-5000 N=15 \
    mp6k_de=--mean mp6k_n=1 mp6k=$(<mp6k+unknown.in) scaffolds
runBESST --orientation rf -c hsapiens-scaffolds.fa -f mp6k.bam -o .
LINKS -f discovar-scaffold.fa -s mpet.fof -d 6000 -k 20 \
    -b discovarlinksMPET6K500z -l 10 -t 10 -m 1 -e 0.8 -z 500
```

Listing S8 MaSuRCA. The script `assemble.sh` is generated by `masurca` itself.

```
./masurca config.txt
./assemble.sh
```

Listing S9 MaSuRCA `config.txt`
https://github.com/bcgsc/abyss-2.0-giab/blob/1.0/masurca/config.txt

```
DATA
PE= 01 400 90 D3_S1_L001_R1_001.bfc.fq.gz D3_S1_L001_R2_001.bfc.fq.gz
PE= 02 400 90 D3_S1_L001_R1_002.bfc.fq.gz D3_S1_L001_R2_002.bfc.fq.gz
PE= 03 400 90 D3_S1_L001_R1_003.bfc.fq.gz D3_S1_L001_R2_003.bfc.fq.gz
PE= 04 400 90 D3_S1_L001_R1_004.bfc.fq.gz D3_S1_L001_R2_004.bfc.fq.gz
...
PE= 32 400 90 D3_S3_L002_R1_001.bfc.fq.gz D3_S3_L002_R2_001.bfc.fq.gz
PE= 33 400 90 D3_S3_L002_R1_002.bfc.fq.gz D3_S3_L002_R2_002.bfc.fq.gz
PE= 34 400 90 D3_S3_L002_R1_003.bfc.fq.gz D3_S3_L002_R2_003.bfc.fq.gz
PE= 35 400 90 D3_S3_L002_R1_004.bfc.fq.gz D3_S3_L002_R2_004.bfc.fq.gz
JUMP= m1 6000 1400 MPHG004-23100079/MPHG004_S3_L003_R1_001.fastq.gz \
    MPHG004-23100079/MPHG004_S3_L003_R2_001.fastq.gz
JUMP= m2 6000 1400 MPHG004-23110109/MPHG004_S3_L003_R1_001.fastq.gz \
    MPHG004-23110109/MPHG004_S3_L003_R2_001.fastq.gz
END

PARAMETERS
GRAPH_KMER_SIZE = auto
USE_LINKING_MATES = 1
LIMIT_JUMP_COVERAGE = 300
CA_PARAMETERS = cgwErrorRate=0.15 ovlMemory=4GB
KMER_COUNT_THRESHOLD = 1
NUM_THREADS = 64
JF_SIZE = 200000000
DO_HOMOPOLYMER_TRIM = 0
END
```

Listing S10 MEGAHIT. The variable `$pe400` is the list of paths to the BFC-corrected, interleaved, and gzipped paired-end FASTQ files.

```
megahit --12 $pe400 --verbose -t 64 --k-list 17,45,73,101,129,157,185,213,241
```

Listing S11 Minia. The largest value of k supported by Minia is 128.

```
minia -in pe400.in -abundance-min auto -kmer-size 128 -nb-cores 64
```

Listing S12 SGA

```
sga preprocess --pe-mode=2 hsapiens.fa.gz >hsapiens.preprocess.fa
sga index -t 64 -a ropebwt hsapiens.preprocess.fa
sga filter -t 64 hsapiens.preprocess.fa
sga fm-merge -t 64 -m 125 hsapiens.preprocess.filter.pass.fa
sga index -d 1000000 -t 64 hsapiens.preprocess.filter.pass.merged.fa
sga rmdup -t 64 hsapiens.preprocess.filter.pass.merged.fa
sga overlap -t 64 -m 125 hsapiens.preprocess.filter.pass.merged.rmdup.fa
sga assemble -m 125 -o hsapiens hsapiens.preprocess.filter.pass.merged.rmdup.asqg.gz
```

Listing S13 SOAPdenovo2

```
SOAPdenovo-127mer all -K 95 -R -p 64 -s hsapiens.config
```

Listing S14 SOAPdenovo2 `hsapiens.config`
https://github.com/bcgsc/abyss-2.0-giab/blob/1.0/soapdenovo/hsapiens.config

```
max_rd_len=250

[LIB]
avg_ins=400
asm_flags=3
rank=1
pair_num_cutoff=3
map_len=32
p=D3_S1_L001_001.bfc.fa.gz
p=D3_S1_L001_002.bfc.fa.gz
p=D3_S1_L001_003.bfc.fa.gz
p=D3_S1_L001_004.bfc.fa.gz
...
p=D3_S3_L002_001.bfc.fa.gz
p=D3_S3_L002_002.bfc.fa.gz
p=D3_S3_L002_003.bfc.fa.gz
p=D3_S3_L002_004.bfc.fa.gz

[LIB]
avg_ins=6000
reverse_seq=1
asm_flags=2
rank=2
pair_num_cutoff=5
map_len=35
p=MPHG004-23100079/MPHG004_S3_L003_001.mp.bfc.fa.gz
p=MPHG004-23110109/MPHG004_S3_L003_001.mp.bfc.fa.gz
p=MPHG004-23100079/MPHG004_S3_L003_001.unknown.bfc.fa.gz
p=MPHG004-23110109/MPHG004_S3_L003_001.unknown.bfc.fa.gz
```

Listing S15 BioNano `hybridScaffold.pl`

```
hybridScaffold.pl -n hsapiens-scaffolds.fa -b EXP_REFINEFINAL1_q.cmap \
    -c hybridScaffold_config_aggressive.xml -B2 -N2 -o bionano \
    -x -y -m all.bnx -q optArguments_human.xml -e AJmother_autoNoise1.err
```

Listing S16 ARCS. The script `makeTSVfile.py` is available online at https://github.com/sarahyeo/giab.

```
arcs -f hsapiens-scaffolds.fa -a human-alignments.fof \
    -s 98 -g 50000 -c 5 -l 5 -m 50-1000 -d 0 -e 30000 -i 16 -v 1
python makeTSVfile.py hsapiens-scaffolds.fa.scaff_s98_c5_original.gv \
    human_c5.tigpair_checkpoint.tsv hsapiens-scaffolds.fa
LINKS -f hsapiens-scaffolds.fa -s empty.fof -b human_c5 -l 5 -a 0.3
```
