# Supplementary material for: ABySS 2.0: resource-efficient assembly of large genomes using a Bloom filter
Source: Genome Res. 2017 May;27(5):768–77. doi: 10.1101/gr.214346.116 (PMC5411771; doi:10.1101/gr.214346.116)

ABySS paired-end pipeline version 1.5.2

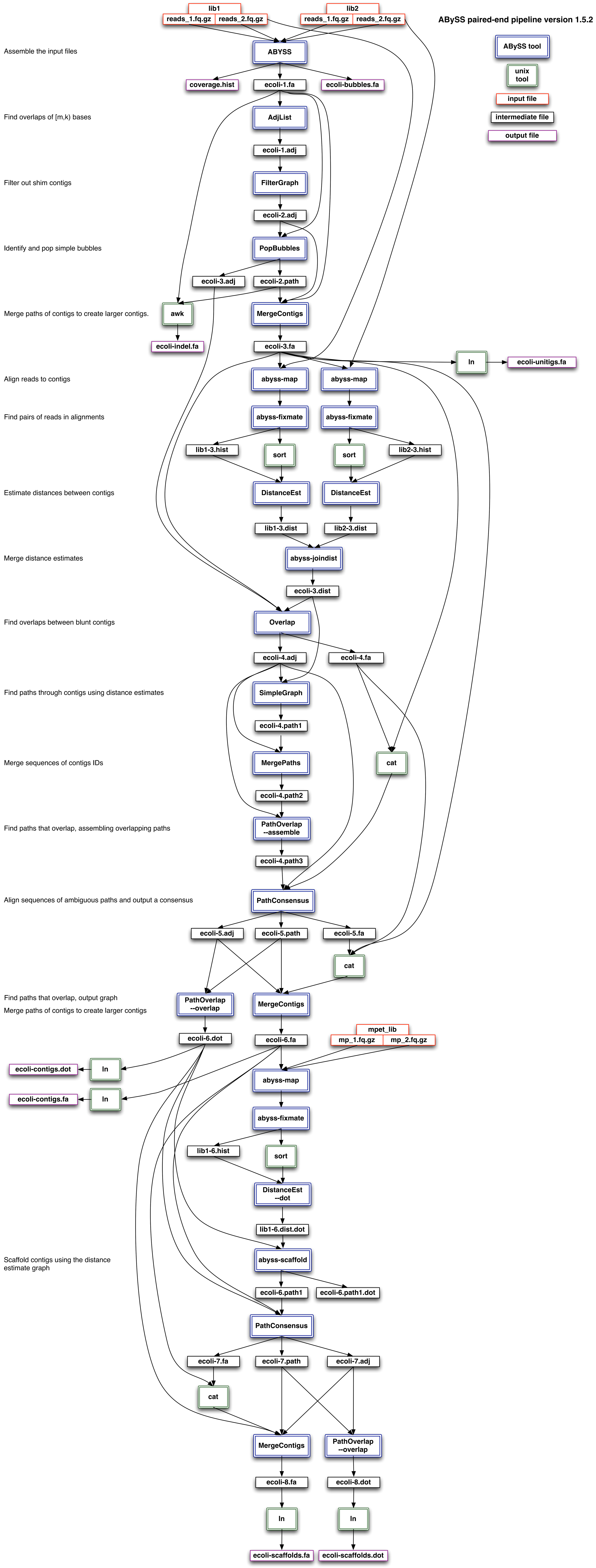

Supplement: Supplemental Material [file supp_gr.214346.116_Supplemental_Archive_1.tar.gz › abyss-bloom-abyss-preview/doc/flowchart.pdf]
