# Supplementary material for: ABySS 2.0: resource-efficient assembly of large genomes using a Bloom filter
Source: Genome Res. 2017 May;27(5):768–77. doi: 10.1101/gr.214346.116 (PMC5411771; doi:10.1101/gr.214346.116)
Supplement: Supplemental Material [file supp_gr.214346.116_Supplemental_Archive_1.tar.gz › abyss-bloom-abyss-preview/doc/flowchart_simplified.pdf]

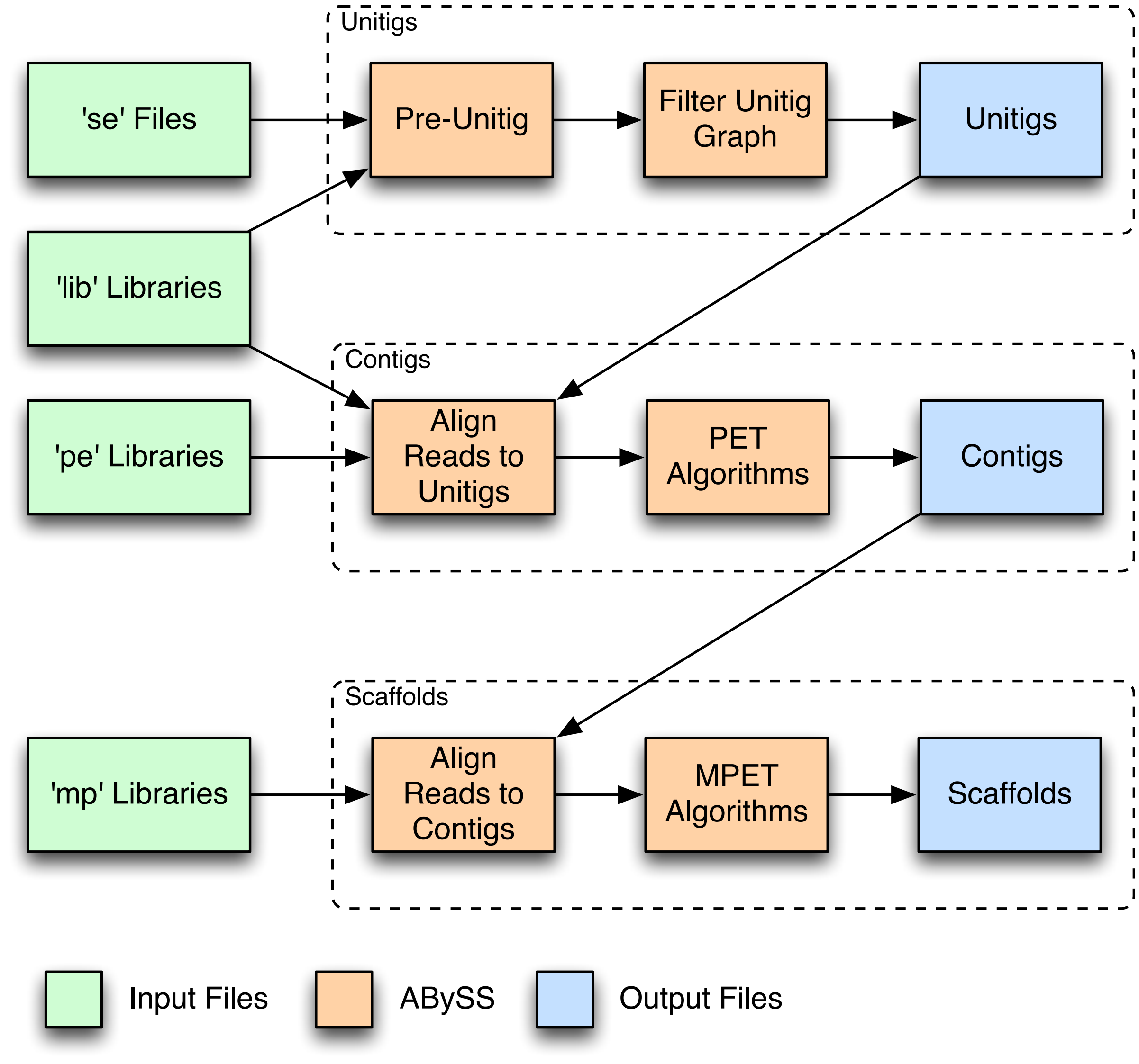

Unitigs
'se' Files
Pre-Unitig
Filter Unitig Graph
Unitigs
'lib' Libraries
Contigs
'pe' Libraries
Align Reads to Unitigs
PET Algorithms
Contigs
Scaffolds
'mp' Libraries
Align Reads to Contigs
MPET Algorithms
Scaffolds
Input Files
ABySS
Output Files
